# Supplementary figures and images for: Turnip mosaic virus P1 suppresses JA biosynthesis by degrading cpSRP54 that delivers AOCs onto the thylakoid membrane to facilitate viral infection
Source: PLoS Pathog. 2021 Dec 1;17(12):e1010108. doi: 10.1371/journal.ppat.1010108 (PMC8668097; doi:10.1371/journal.ppat.1010108)

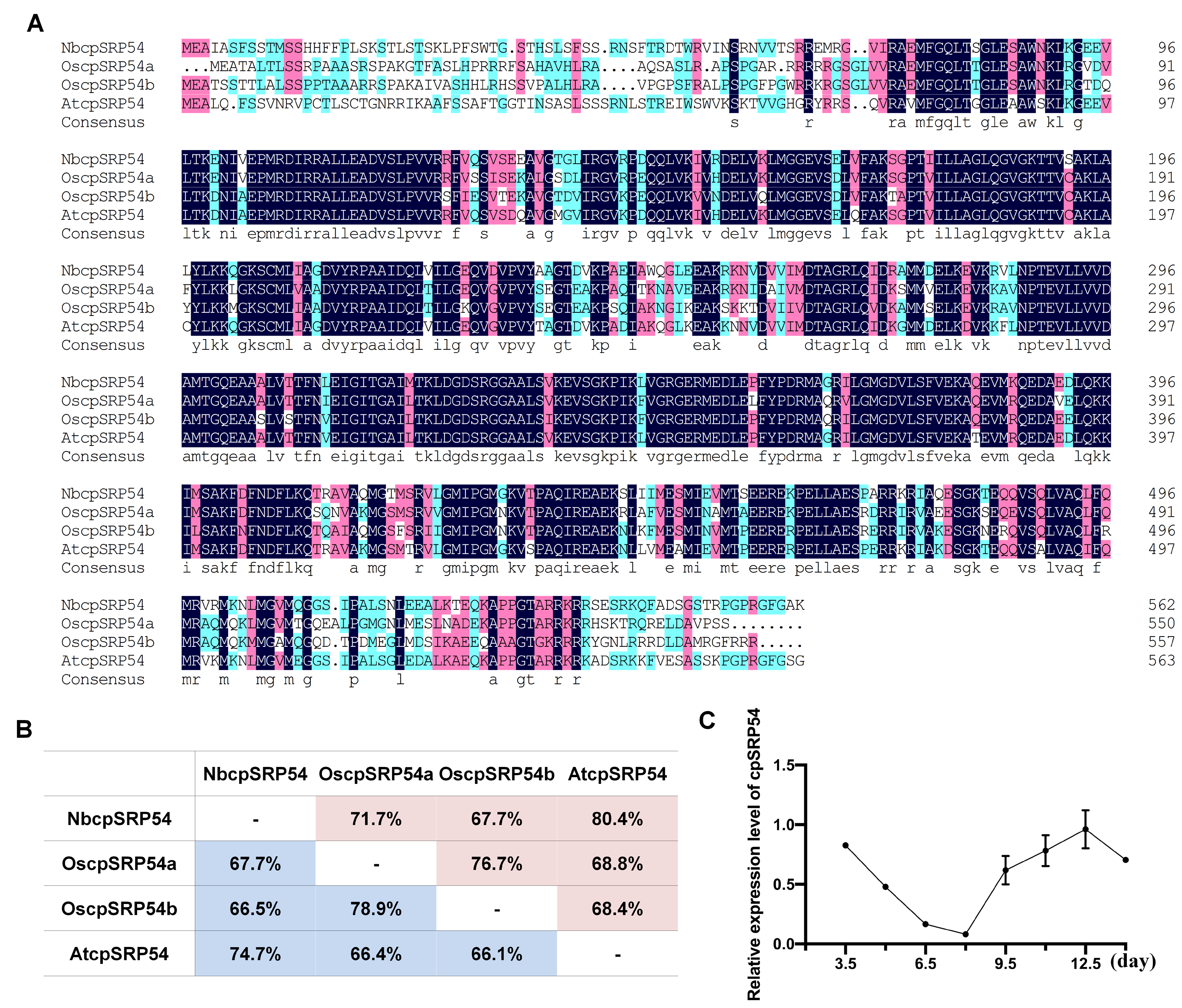

Supplement: S1 Fig — A. Amino acid sequence alignment of cpSRP54s from N. benthamiana, rice and Arabidopsis. B. Amino acid (numbers shadowed with light pink) and nucleotide identities (numbers shadowed with light blue) between the cpSRP54s. C. Quantification of cpSRP54 mRNA levels in TuMV-infected plants within 14 dpi by qRT-PCR analysis. Means ± SD values are from three independent plants per treatment. (TIF) [file ppat.1010108.s001.tif]

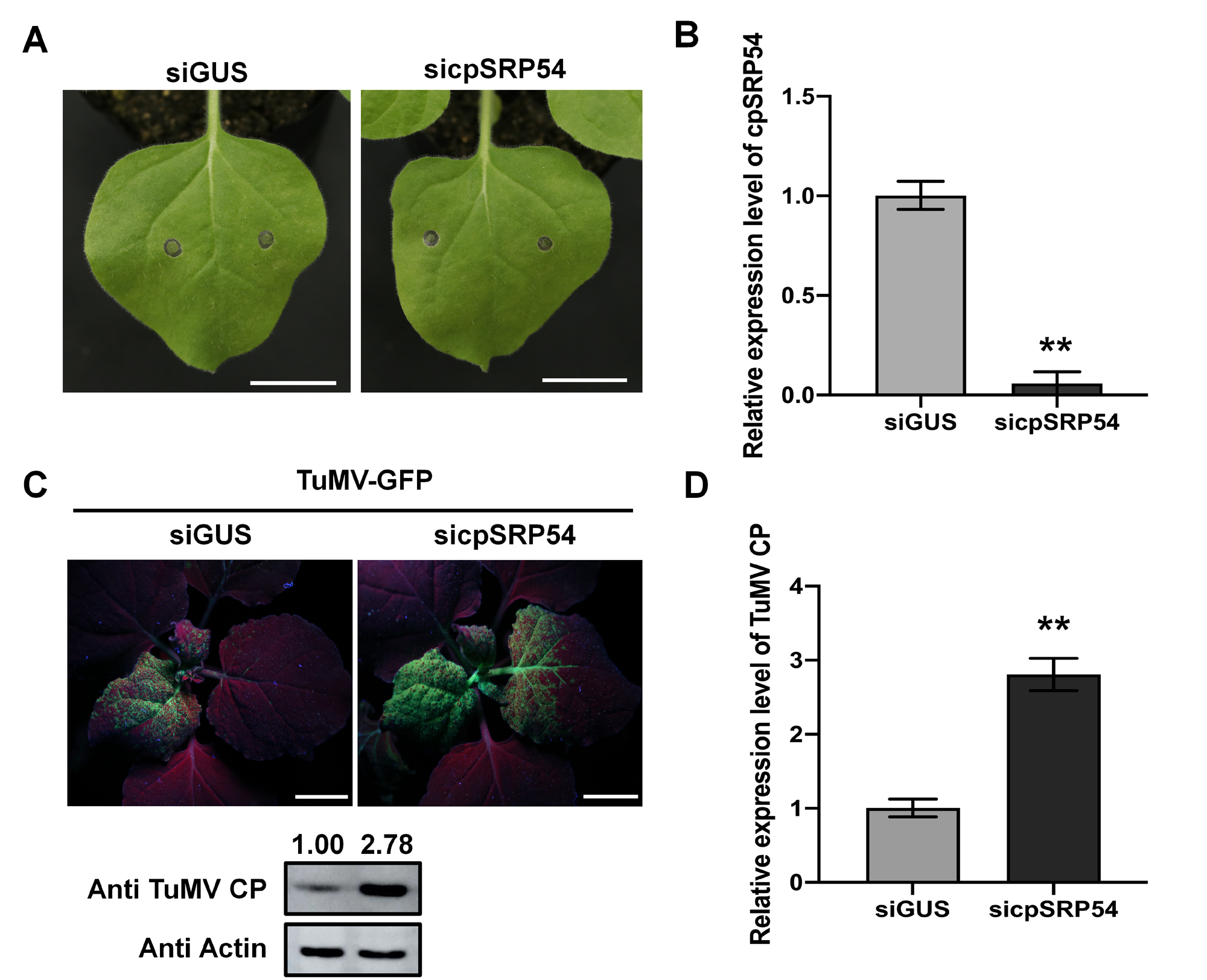

Supplement: S2 Fig — A. Phenotype of leaves inoculated with GUS RNAi construct (as control) and cpSRP54 hairpin RNAi construct at 3 dpi. Silencing of cpSRP54 did not cause obvious chlorosis. B. cpSRP54 mRNA level analysis by qRT-PCR in cpSRP54-silenced plants compared to control plants at 3 dpi. Means ± SD values are from three independent plants per treatment and were normalized against NbActin. **, P<0.01 according to Student’s t-test. C. TuMV-GFP infection in plants pretreated with GUS RNAi construct and cpSRP54 RNAi construct. Plants were photographed under UV at 7 dpi. Viral CP accumulation in systemic leaves was determined by WB. Actin served as a loading control. This experiment was repeated at least three times, and one representative result is shown. The protein levels were quantified by ImageJ. D. Relative viral RNA levels quantified by qRT-PCR. Means ± SD values are from three independent plants per treatment. **, P<0.01 according to Student’s t-test. (TIF) [file ppat.1010108.s002.tif]

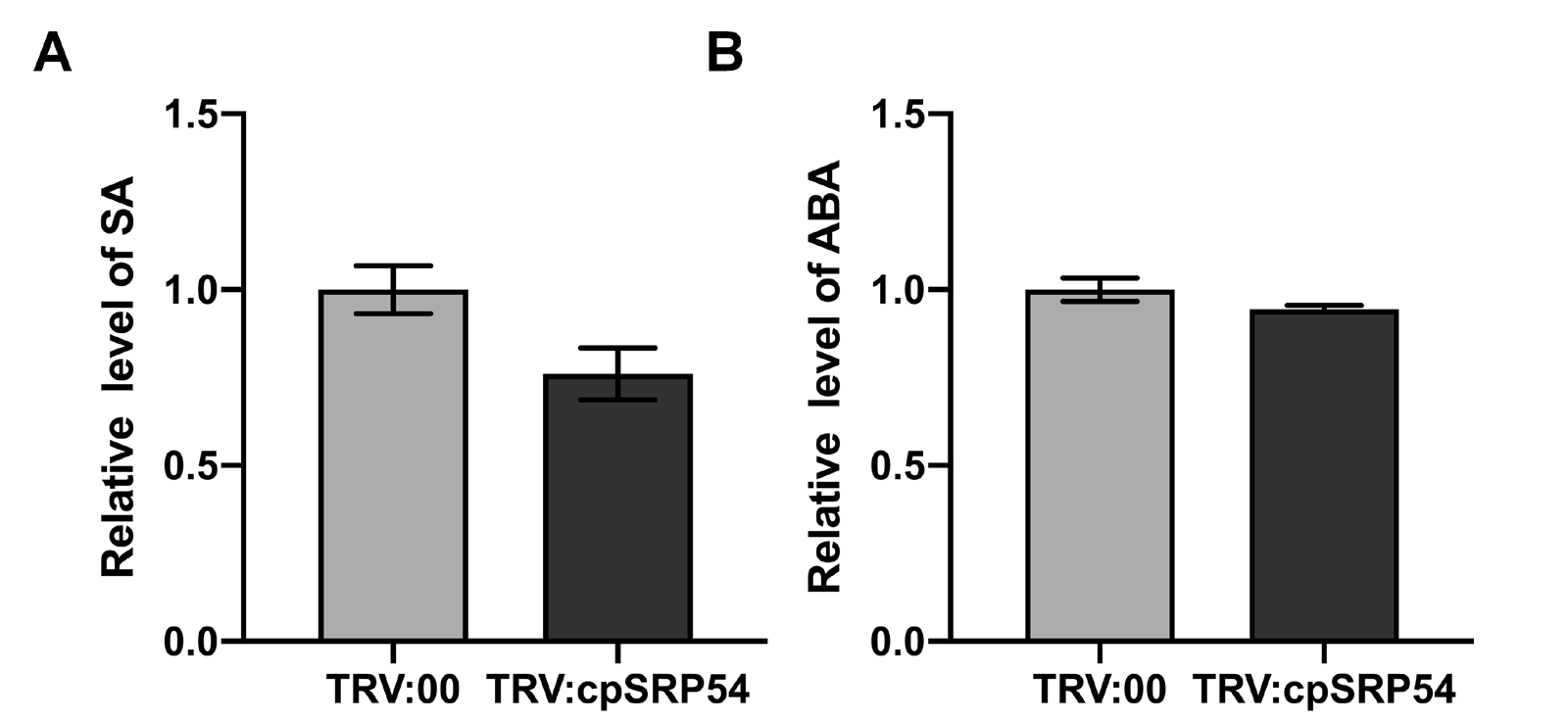

Supplement: S3 Fig — (TIF) [file ppat.1010108.s003.tif]

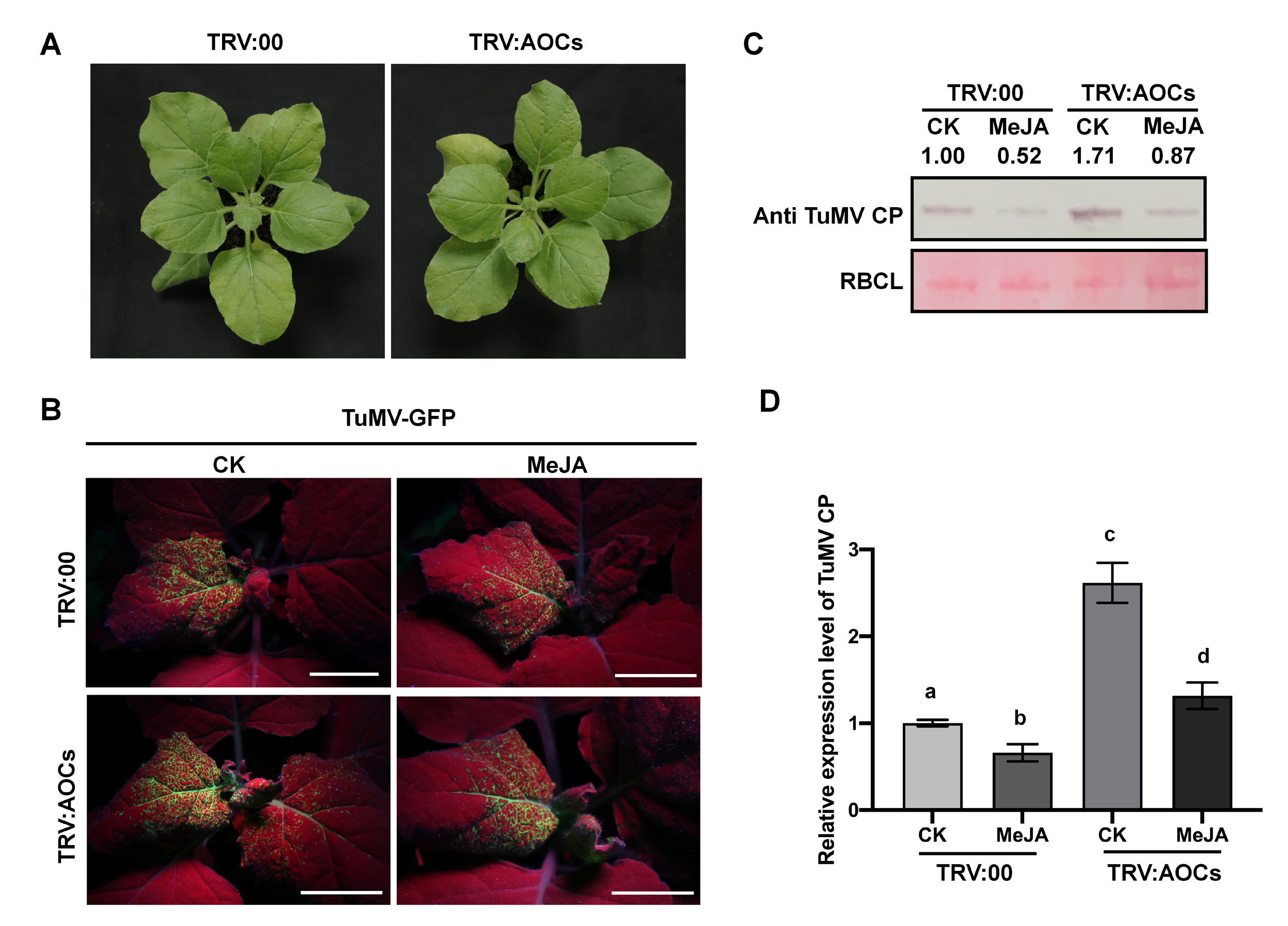

Supplement: S4 Fig — A. Phenotype in TRV:00 and TRV:AOCs treated plants at 12 dpi. B. Effect of MeJA treatment on TuMV-infection in plants inoculated with TRV:00 or TRV:AOCs at 7 dpi. Plants were photographed under UV light. Bars, 2 cm. C. Accumulation of viral CP protein quantified by WB. Ponceau S-stained RBCL was used as a loading control. Tests were performed independently three times with similar results. D. Quantification of viral RNA levels by qRT-PCR. Means ± SD values are from three independent plants per treatment. **, P<0.01, *, P<0.05 according to Student’s t-test. (TIF) [file ppat.1010108.s004.tif]

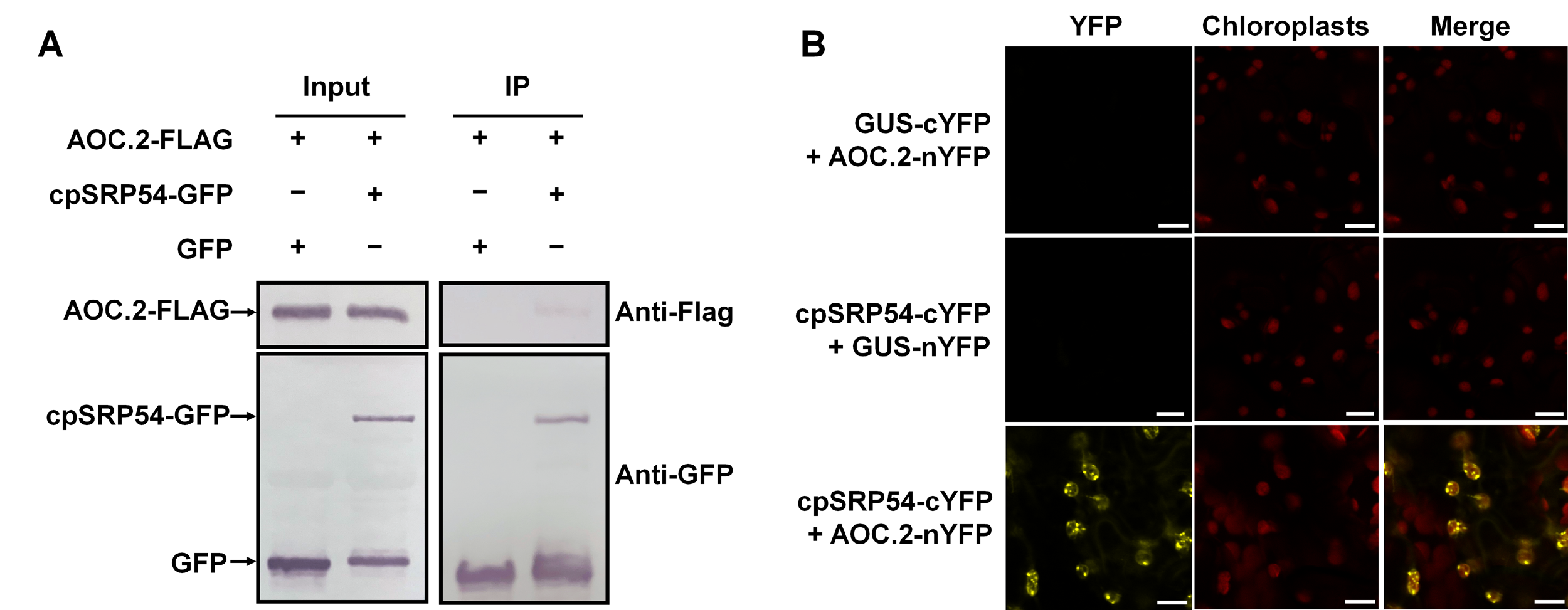

Supplement: S5 Fig — Scale bar, 10 μm. (TIF) [file ppat.1010108.s005.tif]

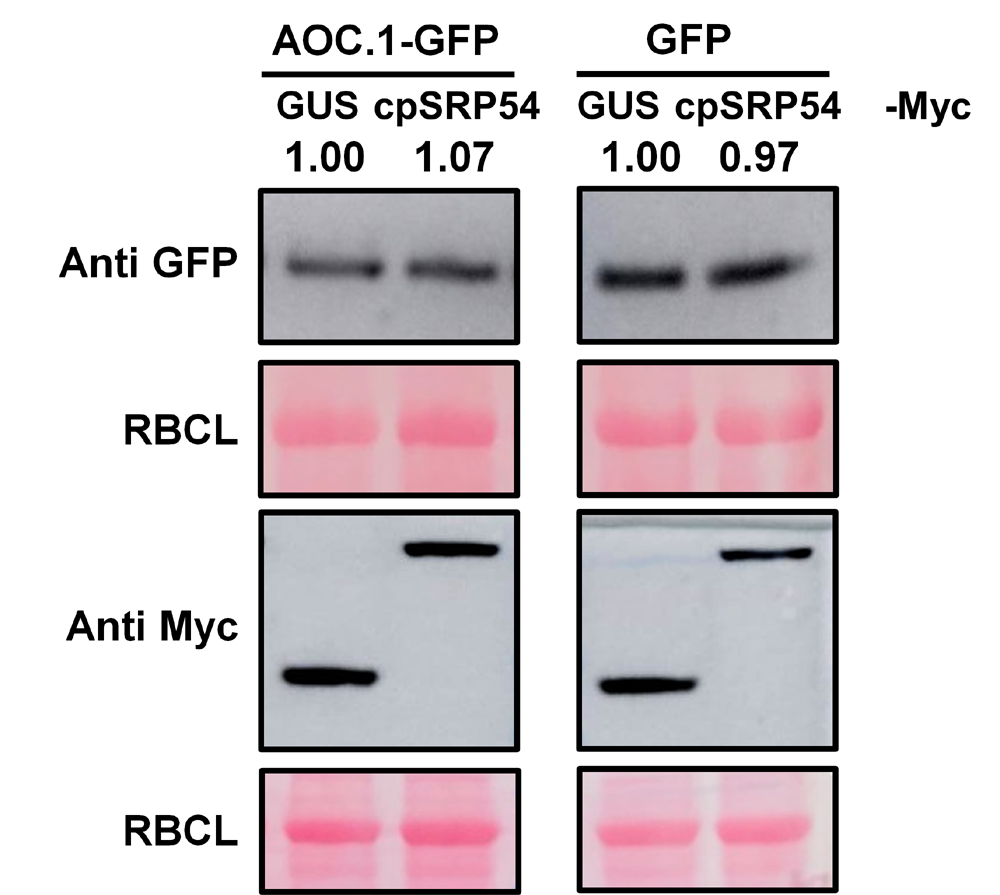

Supplement: S6 Fig — GUS-Myc and empty GFP were used as controls. Ponceau S-stained RBCL was used as a loading control. The protein levels were quantified by ImageJ. Tests were performed independently three times. (TIF) [file ppat.1010108.s006.tif]

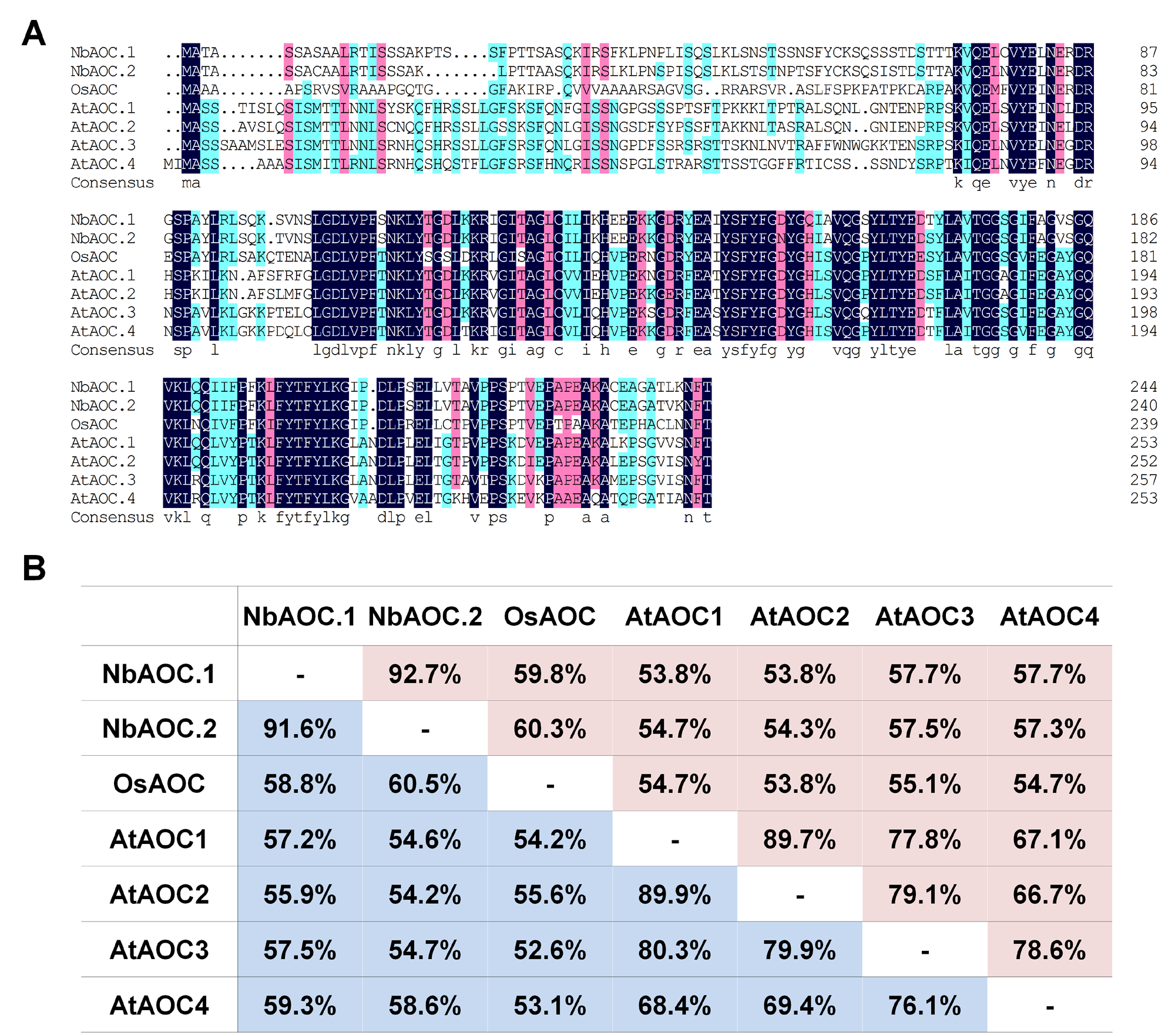

Supplement: S7 Fig — A. Amino acid sequence alignment of AOCs from N. benthamiana, rice and Arabidopsis. B. Amino acid (numbers shadowed with light pink) and nucleotide identities (numbers shadowed with light blue) among NbAOC.1, NbAOC.2, OsAOC, AtAOC1, AtAOC2, AtAOC3 and AtAOC4. (TIF) [file ppat.1010108.s007.tif]

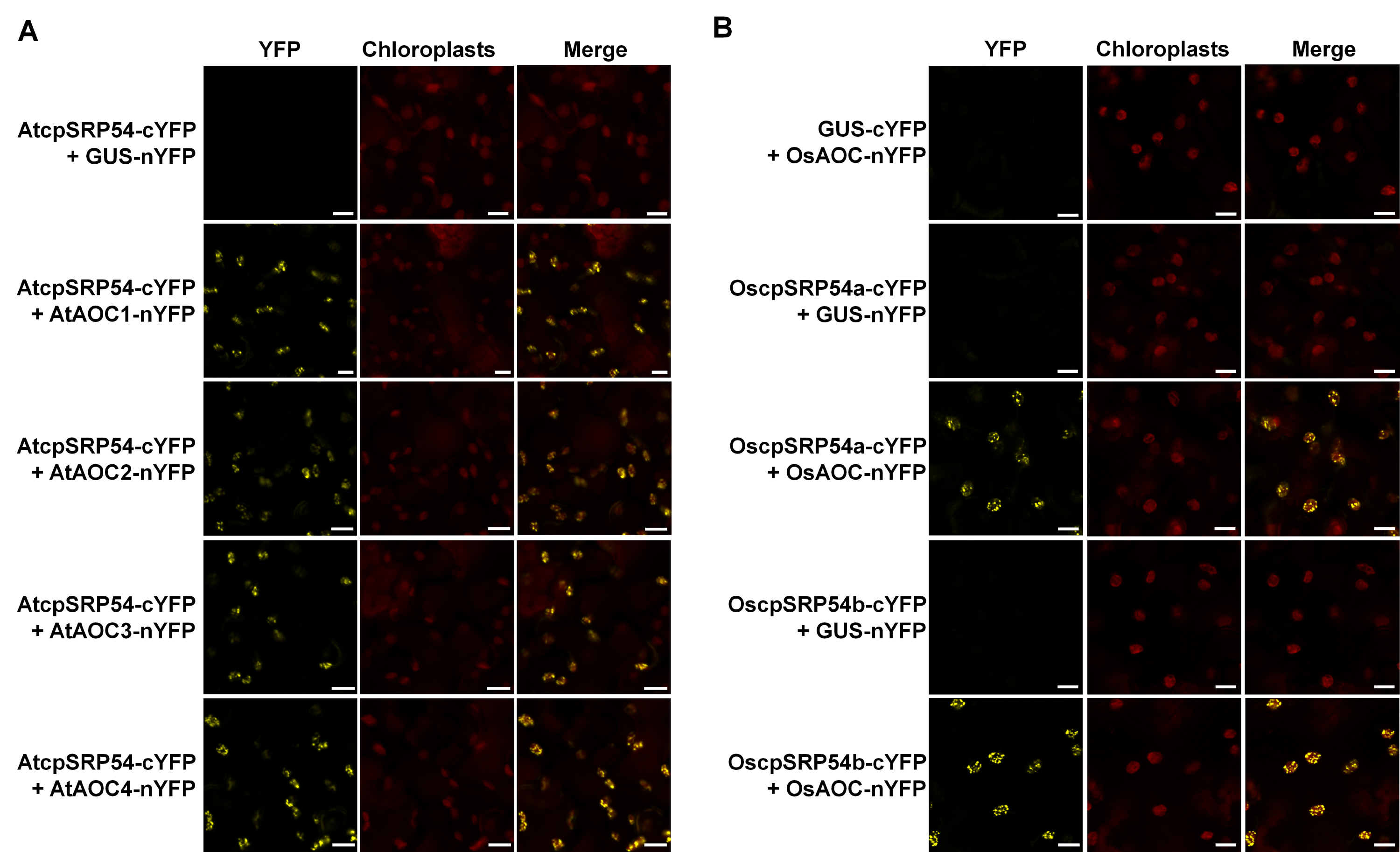

Supplement: S8 Fig — cpSRP54s and AOCs were respectively fused to the C-terminal (cYFP) and N-terminal (nYFP) half of YFP. Confocal imaging was performed at 2 dpi. Scale bar, 10 μm. (TIF) [file ppat.1010108.s008.tif]

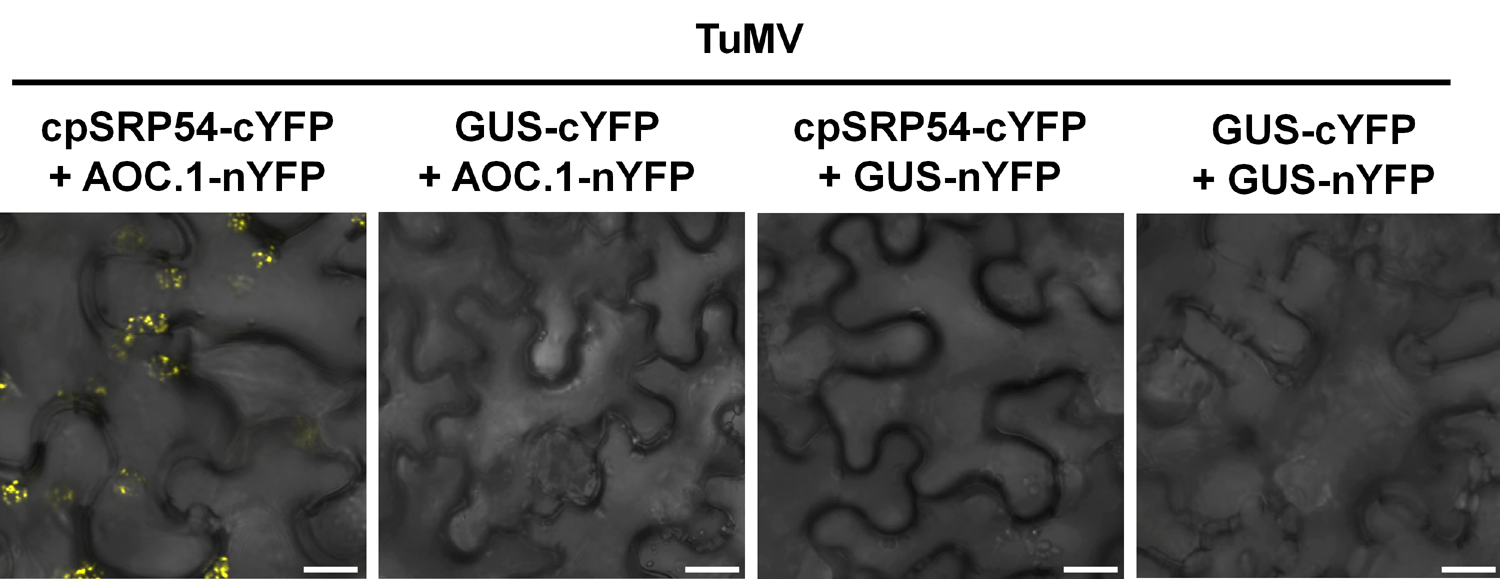

Supplement: S9 Fig — Scale bar, 10 μm. (TIF) [file ppat.1010108.s009.tif]

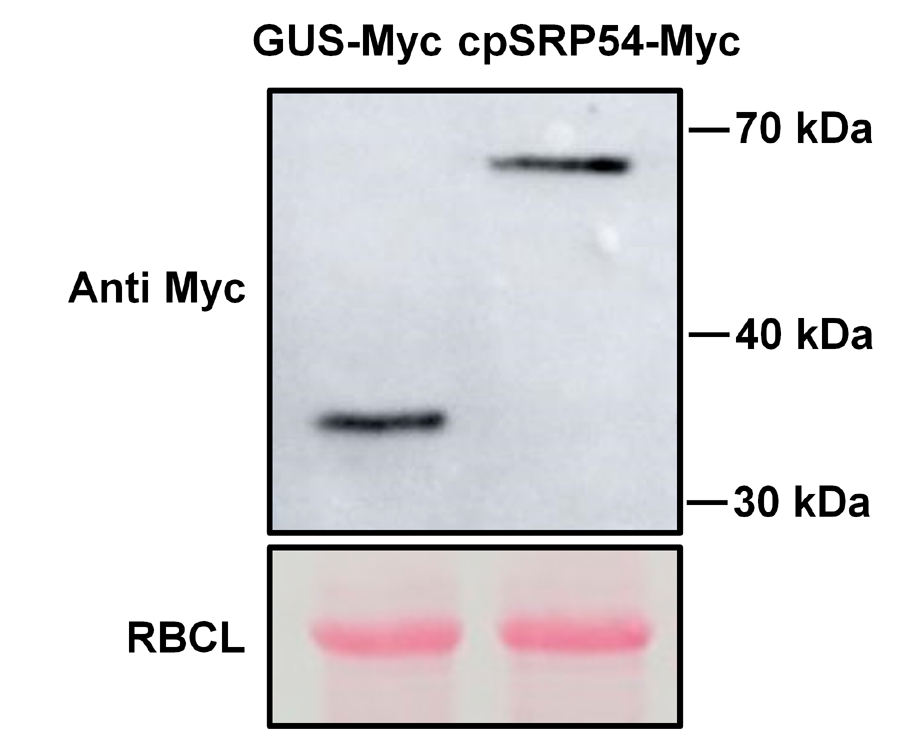

Supplement: S10 Fig — (TIF) [file ppat.1010108.s010.tif]

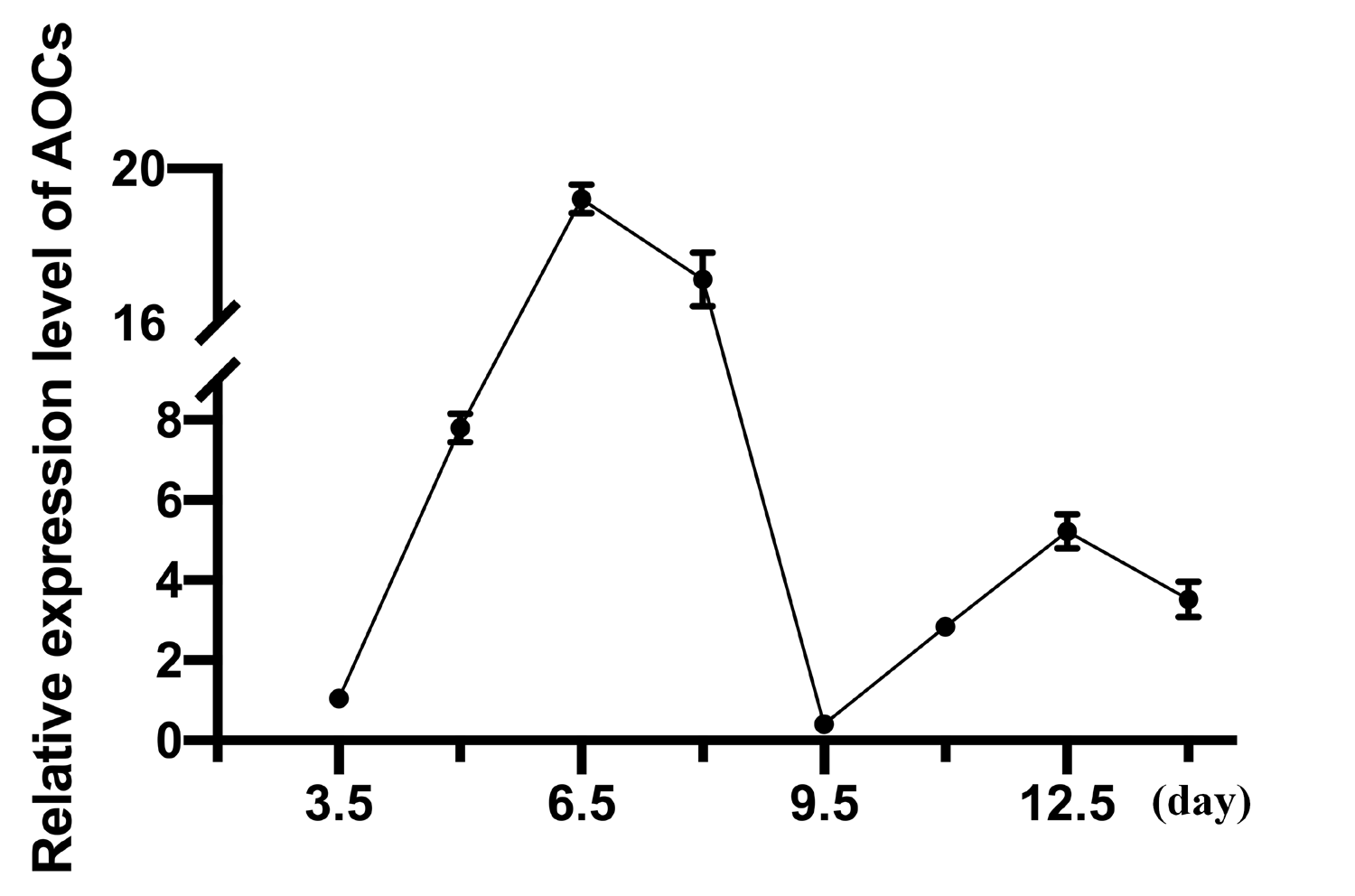

Supplement: S11 Fig — Means ± SD values are from three independent plants per treatment. (TIF) [file ppat.1010108.s011.tif]

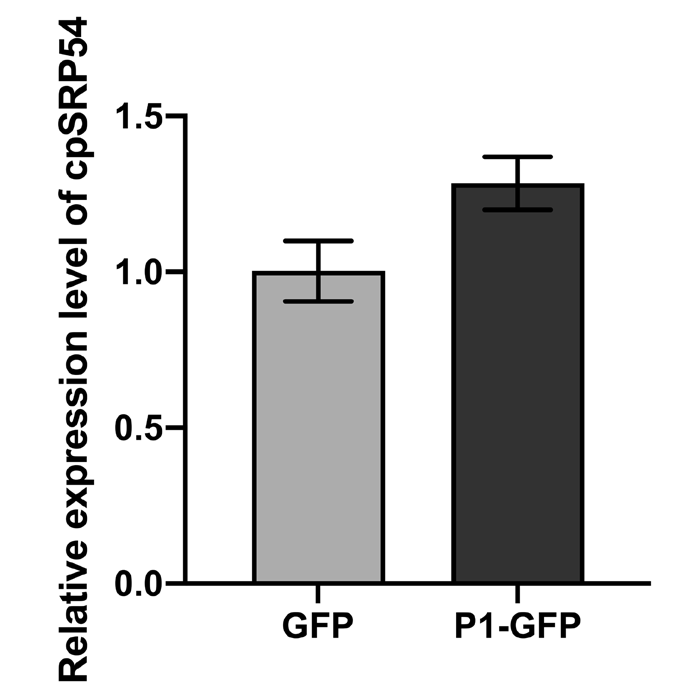

Supplement: S12 Fig — Relative expression level of cpSRP54 in N. benthamiana plants transiently expressed GFP or P1-GFP at 2 dpi. Bars represent the standard errors of the means from three biological repeats. NbActin was used as the internal control. (TIF) [file ppat.1010108.s012.tif]

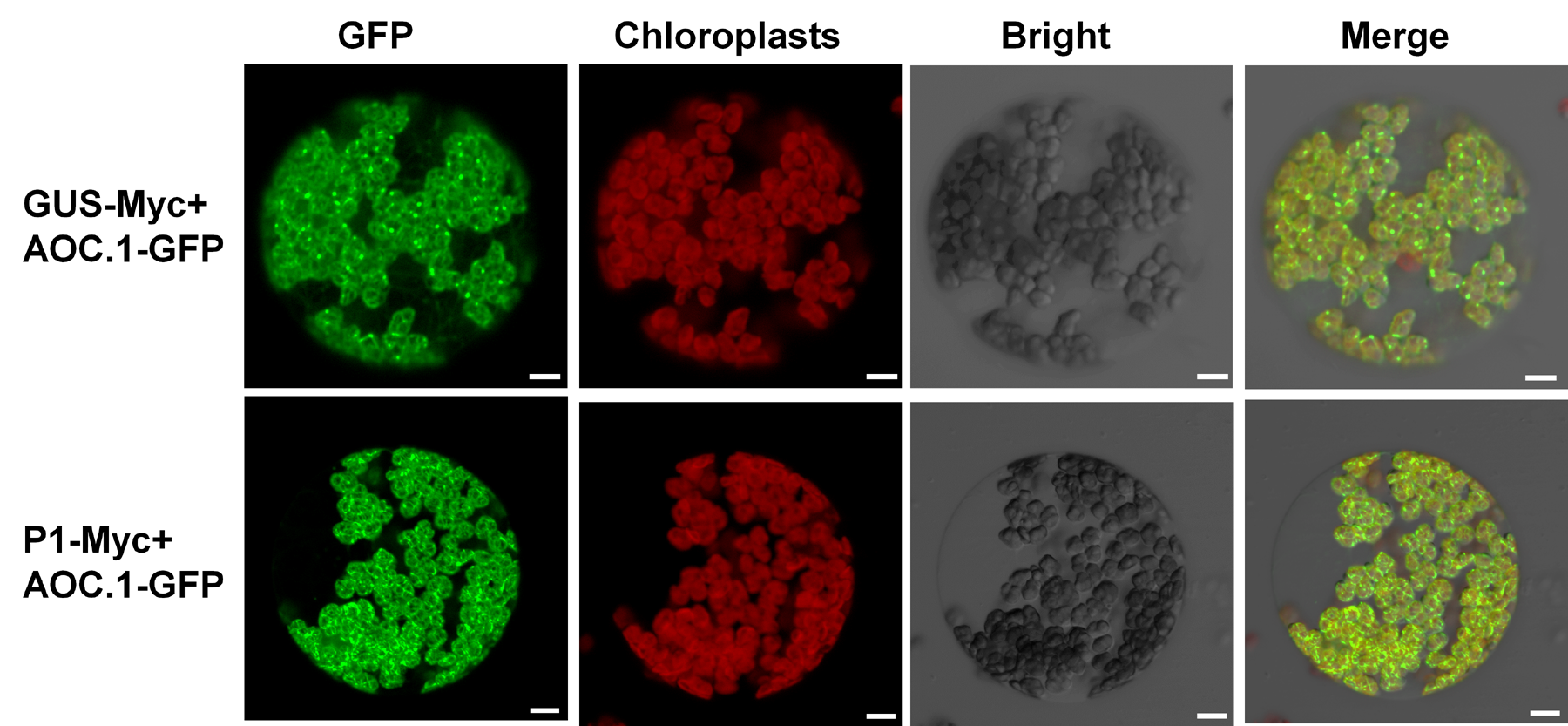

Supplement: S13 Fig — Bars, 10 μm. (TIF) [file ppat.1010108.s013.tif]

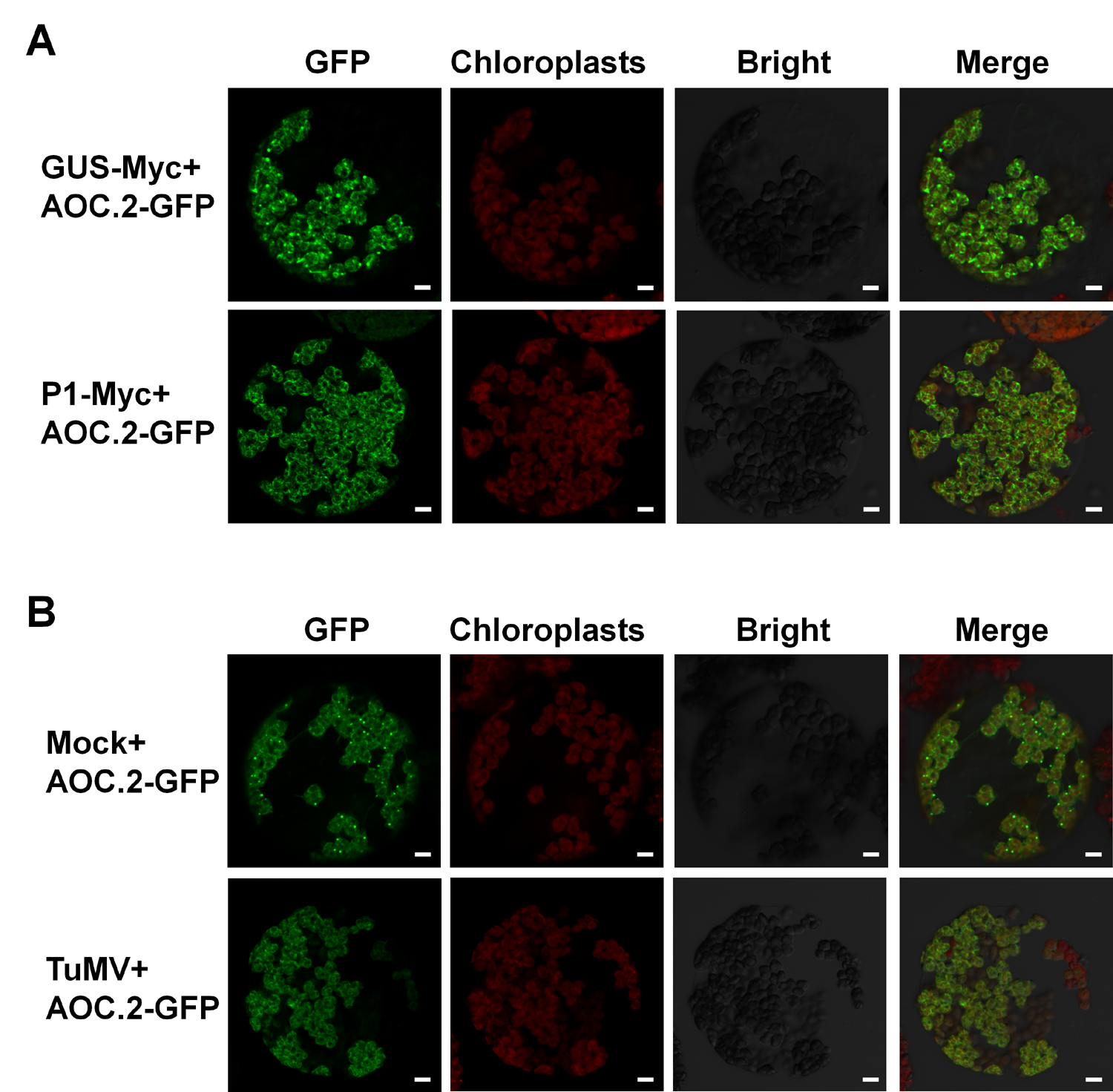

Supplement: S14 Fig — (TIF) [file ppat.1010108.s014.tif]

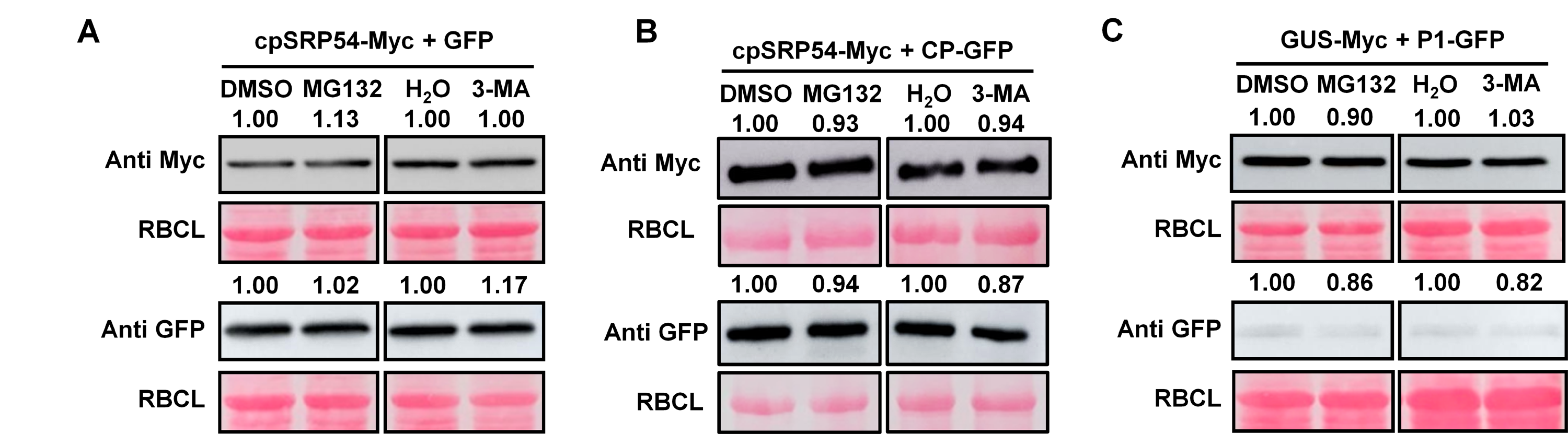

Supplement: S15 Fig — A and B. Accumulation of cpSRP54-Myc was not affected by empty GFP (A), or CP-GFP (B) whether treated with MG132 and 3-MA or not. B. P1-GFP did not affect accumulation of GUS-Myc, whether treated with MG132 and 3-MA or not. (TIF) [file ppat.1010108.s015.tif]

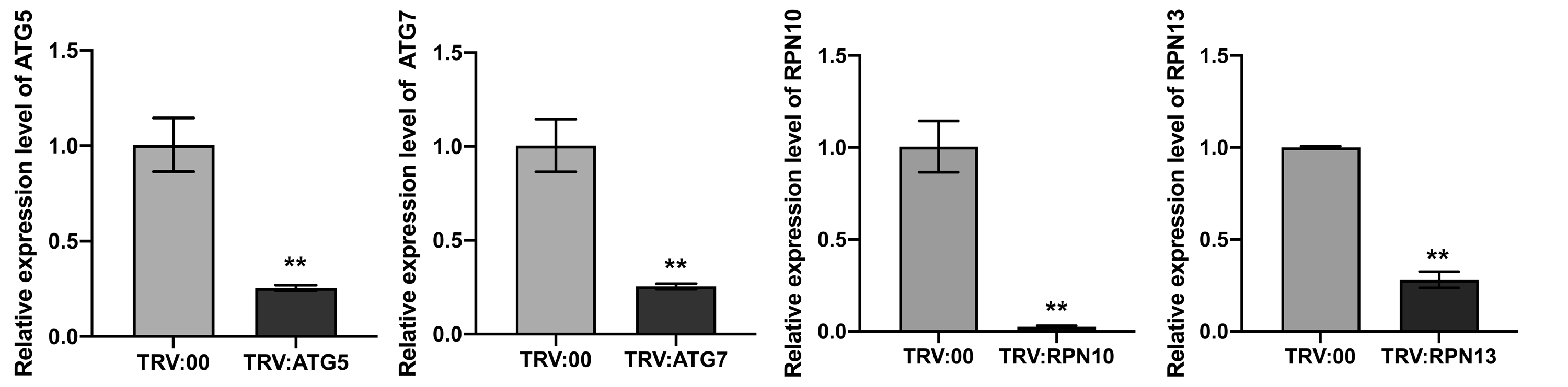

Supplement: S16 Fig — The leaf samples were harvested at 14 dpi. Values represent the Means ± SD. **, P<0.01 according to Student’s t-test. (TIF) [file ppat.1010108.s016.tif]

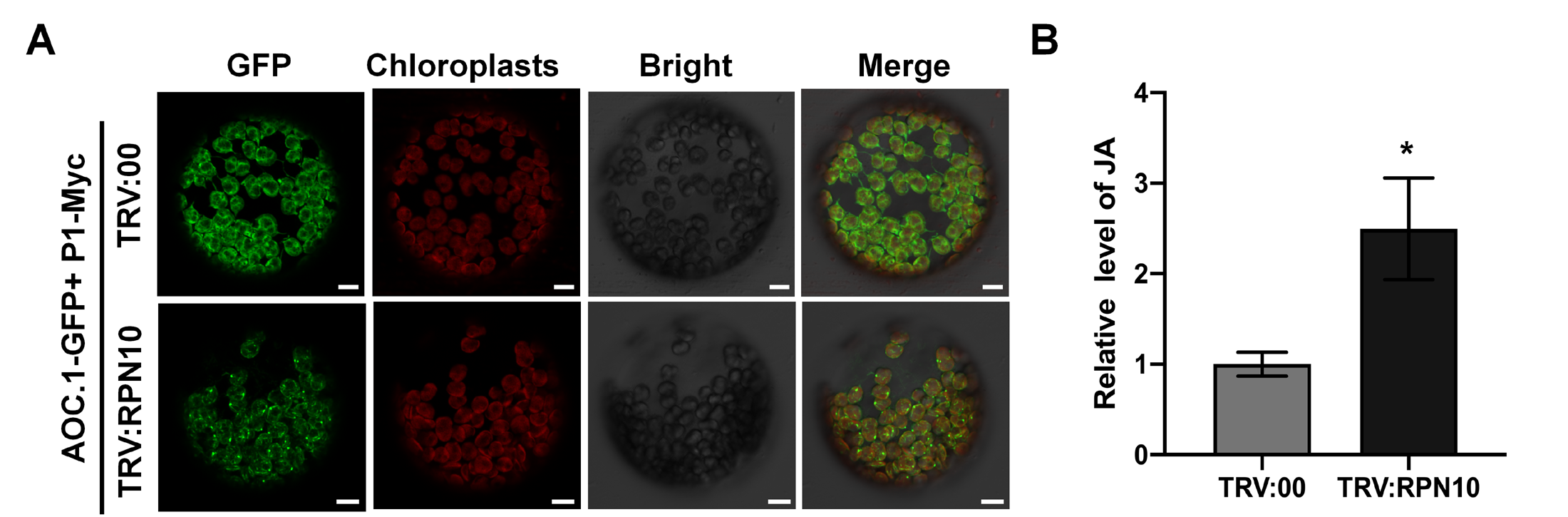

Supplement: S17 Fig — (A) Confocal microscopy analysis showing the localization of AOC.1-GFP transiently expressed in TRV:00 or TRV:RPN10 treated plants when co-expressed with P1-Myc at 3 dpi. (B) The relative JA level in the above plants. Three independent replicates each containing three biological repeats were used for hormone quantification. *, P<0.05 according to Student’s t-test. (TIF) [file ppat.1010108.s017.tif]

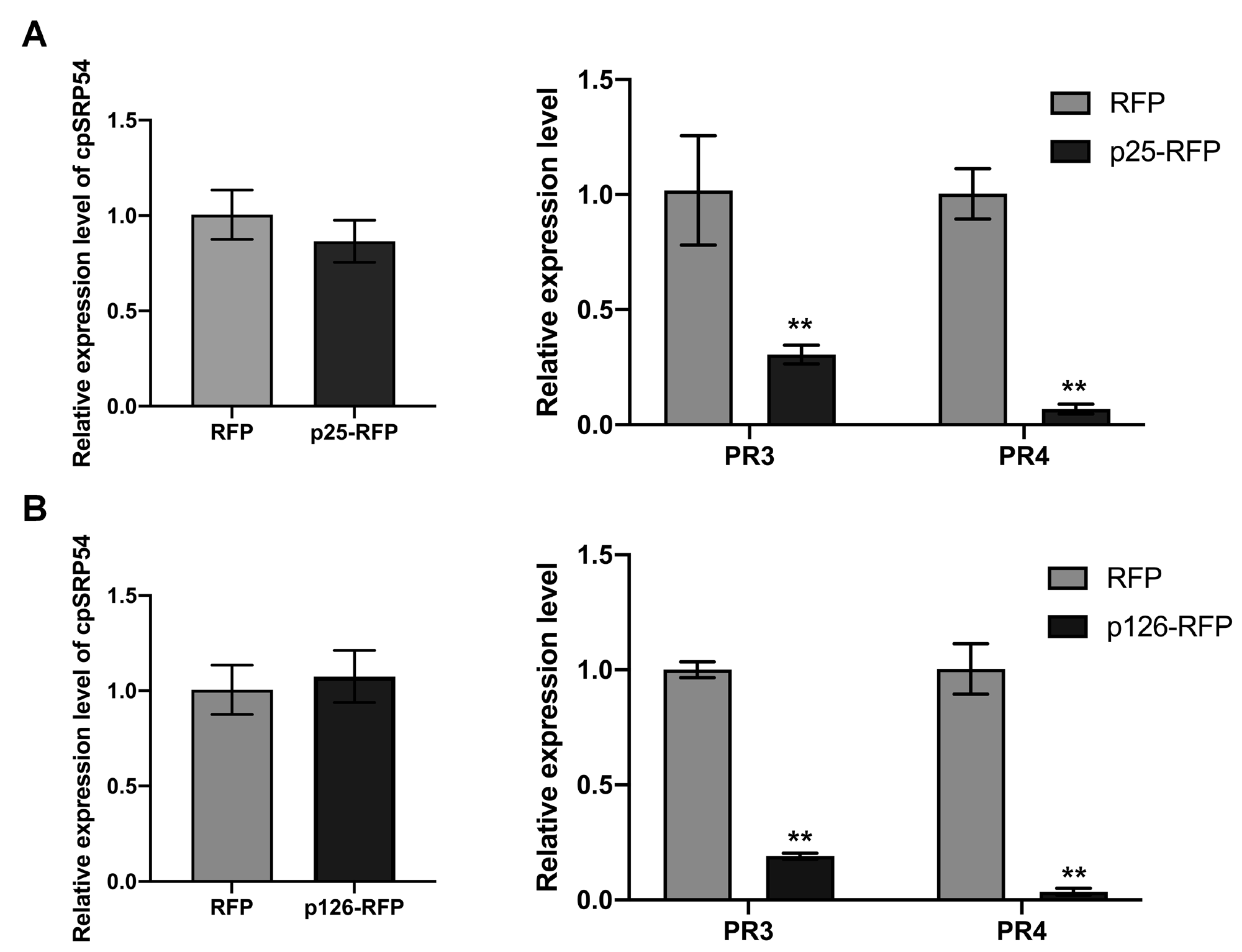

Supplement: S18 Fig — (TIF) [file ppat.1010108.s018.tif]

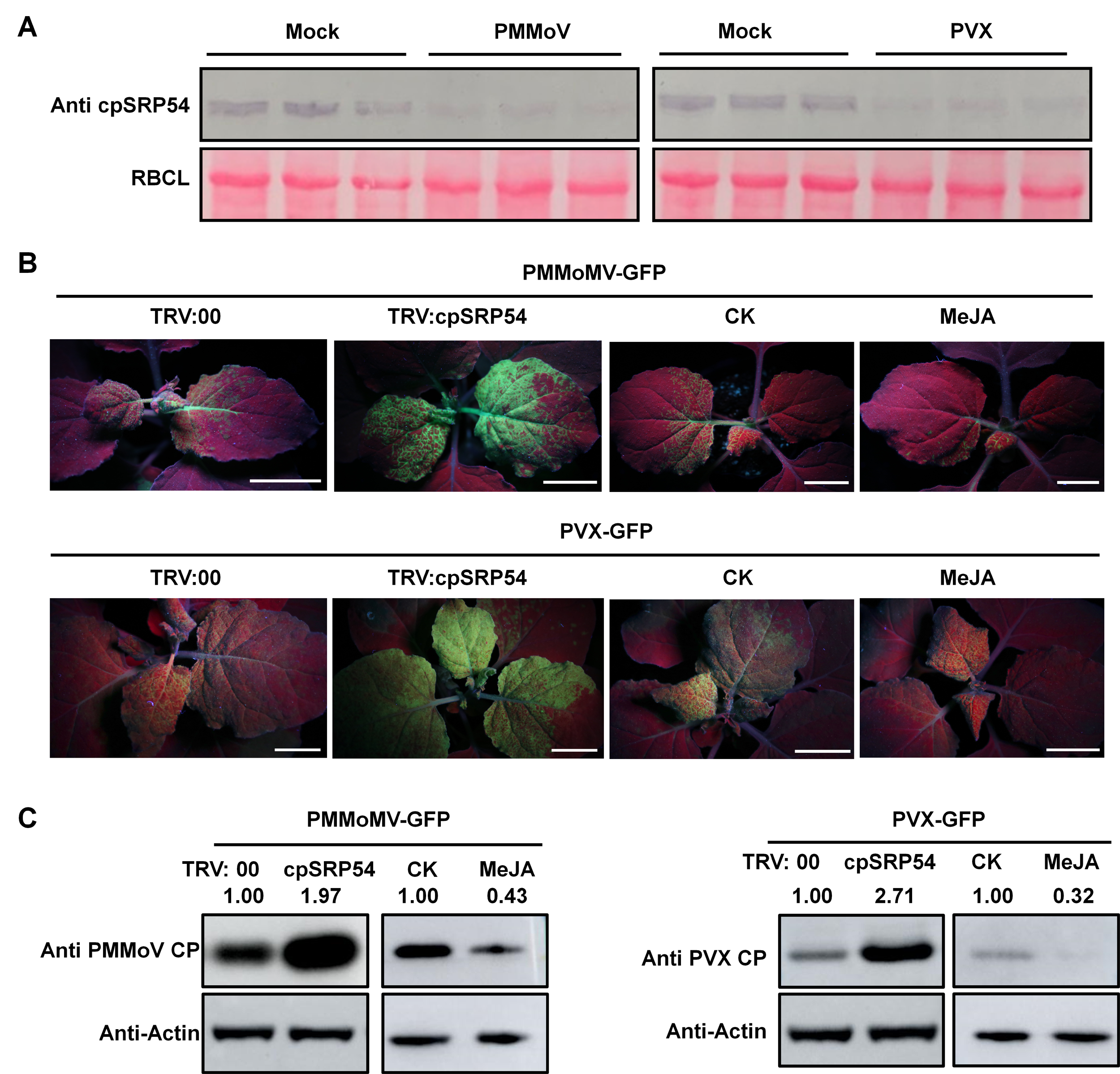

Supplement: S19 Fig — A. The accumulation of cpSRP54 proteins in PMMoV or PVX-infected N. benthamiana was determined by WB. B. PMMoV-GFP or PVX-GFP were inoculated onto cpSRP54-silenced (TRV:cpSRP54), non-silenced, CK (0.1% ethanol) treated wild type and MeJA (50 μM) treated wild type plants. GFP fluorescence on the newly-emerged leaves indicate systemic infection by viruses at 7 dpi. Plants were photographed under UV light. C. PMMoV and PVX CP antibody were used to detect the accumulation of PMMoV-GFP and PVX-GFP, respectively, in plants at 7 dpi. The protein levels were quantified by ImageJ and normalized against actin protein levels. These experiments are the representatives of three independent biological experiments with similar results. (TIF) [file ppat.1010108.s019.tif]

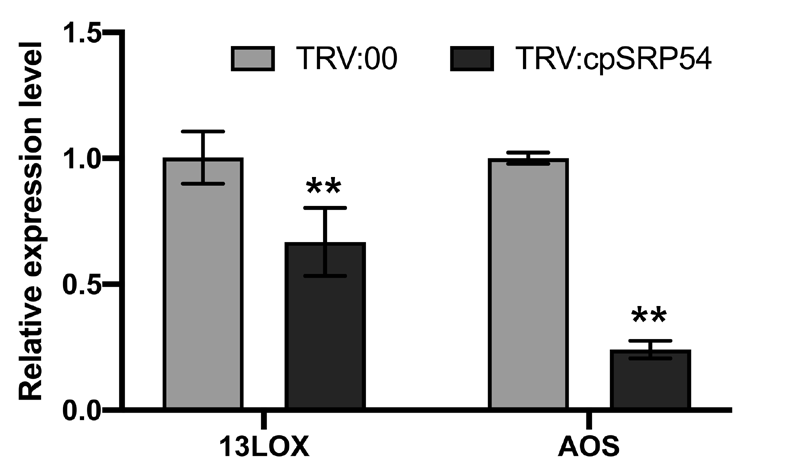

Supplement: S20 Fig — (TIF) [file ppat.1010108.s020.tif]

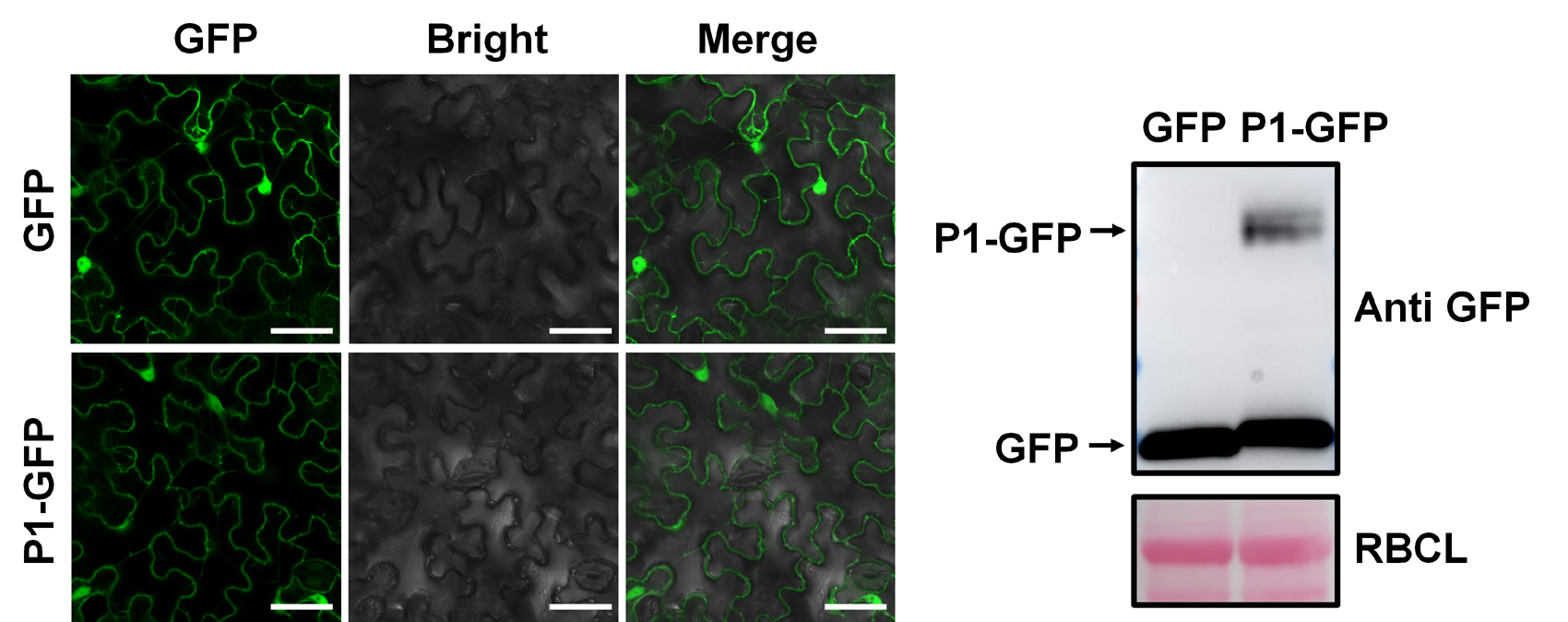

Supplement: S21 Fig — (TIF) [file ppat.1010108.s021.tif]
