# Supplementary material for: Turnip mosaic virus P1 suppresses JA biosynthesis by degrading cpSRP54 that delivers AOCs onto the thylakoid membrane to facilitate viral infection
Source: PLoS Pathog. 2021 Dec 1;17(12):e1010108. doi: 10.1371/journal.ppat.1010108 (PMC8668097; doi:10.1371/journal.ppat.1010108)
Supplement: S1 Table — (DOCX) [file ppat.1010108.s022.docx]

S1 Table Primers used for analysis

| **Name** | **Primers (5'-3')** | **Description** |
| --- | --- | --- |
| NbcpSRP54-ORF-f | cgacgacaagaccgtcaccATGGAAGCCATTGCTTCT | forward primer for pCV:cpSRP54/pDHB1-cpSRP54 by lic |
| NbcpSRP54-ORF-r | ggaggagaagagccgtcgGTTCTTG GCCCCAAAACC | reverse primer for pCV:cpSRP54/pDHB1-cpSRP54 by lic |
| NbcpSRP54-300nt-f | cgacgacaagaccgtGGGACTGGCTTGATTCGA | forward primer for TRV:NbcpSRP54 by lic |
| NbcpSRP54-300nt-r | gaggagaagagccgtcgTGCATAAACA GGCACATC | reverse primer for TRV:NbcpSRP54 by lic |
| siNbcpSRP54(BamHI/NcoI) | ggatcccatggCCCACCATAATACTATTA | forward primer for *NbcpSRP54* RNAi harpin |
| siNbcpSRP54(XbaI/XhoI) | tctagactcgagTGCATAAACAGGCACATC | reverse primer for *NbcpSRP54* RNAi harpin |
| siGus(BamHI/NcoI) | ggatcccatggATGTTACGTCCTGTAGAAA | forward primer for *Gus* RNAi harpin |
| siGus(XbaI/XhoI) | tctagactcgagGATCGTTAAAACTGCCTGG | reverse primer for *Gus* RNAi harpin |
| NbAOC.1-ORF-f | cgacgacaagaccgtcaccATGGCCACTGCCTCCTCAG | forward primer for pCV:NbAOC.1/ pPR3-AOC.1 by lic |
| NbAOC.1-ORF-r | gaggagaagagccgtcgATTAGTGAAA TTTTTCAG | reverse primer for pCV:NbAOC.1/ pPR3-AOC.1 by lic |
| NbAOC.2-ORF-f | cgacgacaagaccgtcaccATGGCCACTGCCCCCTCC | forward primer for pCV:NbAOC.2/ pPR3-AOC.2 by lic |
| NbAOC.2-ORF-r | gaggagaagagccgtcgATTGGTGAAATTTTTCAC | reverse primer for pCV:NbAOC.2/ pPR3-AOC.2 by lic |
| NbAOCs-300nt-f | cgacgacaagaccgtTGGCCACTG CCTCCTCAGCCTC | forward primer for TRV:NbAOCs by lic |
| NbAOCs-300nt-r | gaggagaagagccgtcgGACAGACTTTTGGCTCAAGC | reverse primer for TRV:NbAOCs by lic |
| OscpSRP54a-ORF-f | cgacgacaagaccgtcaccATGGAGGCCACGGCGCTCACG | forward primer for pCV:OscpSRP54a-nYFP by lic |
| OscpSRP54a-ORF-r | gaggagaagagccgtcgAGAACTAGGGACTGCATCCAGC | reverse primer for pCV:OscpSRP54a-nYFP by lic |
| OscpSRP54b-ORF-f | cgacgacaagaccgtcaccATGGAGGCCACCAGTAGT | forward primer for pCV:OscpSRP54b-nYFP by lic |
| OscpSRP54b-ORF-r | gaggagaagagccgtcgTCGTCGACGG AAACCGCGCAT | reverse primer for pCV:OscpSRP54b-nYFP by lic |
| AtcpSRP54-ORF-f | cgacgacaagaccgtcaccATGGAGGCTCTTCAATTT | forward primer for pCV:AtcpSRP54-nYFP by lic |
| AtcpSRP54-ORF-r | gaggagaagagccgtcgGTTACCAGAGCCGAAGCC | reverse primer for pCV:AtcpSRP54-nYFP by lic |
| OsAOC-ORF-f | cgacgacaagaccgtcaccATGGCCGCCGCCGCCCCCTC | forward primer for pCV:OsAOC-cYFP by lic |
| OsAOC-ORF-r | gaggagaagagccgtcgGTTGGTGAAGTTGTTGAGGC | reverse primer for pCV:OsAOC-cYFP by lic |
| AtAOC1-ORF-f | cgacgacaagaccgtcaccATGGCTTCTTCTACAATC | forward primer for pCV:AtAOC1-cYFP by lic |
| AtAOC1-ORF-r | gaggagaagagccgtcgATTTGTAAAGTTGCTTAC | reverse primer for pCV:AtAOC1-cYFP by lic |
| AtAOC2-ORF-f | cgacgacaagaccgtcaccATGGCTTCTTCAGCAGTG | forward primer for pCV:AtAOC2-cYFP by lic |
| AtAOC2-ORF-r | gaggagaagagccgtcgGTTGGTATAGTTACTTAT | reverse primer for pCV:AtAOC2-cYFP by lic |
| AtAOC3-ORF-f | cgacgacaagaccgtcaccATGGCTTCTTCTTCTGCT | forward primer for pCV:AtAOC3-cYFP by lic |
| AtAOC3-ORF-r | gaggagaagagccgtcgATTAGTAAAGTTACTTATAAC | reverse primer for pCV:AtAOC3-cYFP by lic |
| AtAOC4-ORF-f | cgacgacaagaccgtcaccATGATCATGGCTTCTTCT | forward primer for pCV:AtAOC4-cYFP by lic |
| AtAOC4-ORF-r | gaggagaagagccgtcgATTAGTAAAGTTAGCGAT | reverse primer for pCV:AtAOC4-cYFP by lic |
| TuMV P1-ORF-f | cgacgacaagaccgtcaccATGGCAGCAGTTACATTCGC | forward primer for pCV:P1 by lic |
| TuMV P1-ORF-r | gaggagaagagccgtcgACTAAAGTGC ACAATCTTGT | reverse primer for pCV:P1 by lic |
| PMMoV p126-ORF-f | cgacgacaagaccgtcaccATGGCTTACACACAACAA | forward primer for pCV:p126 by lic |
| PMMoV p126-ORF-r | gaggagaagagccgtcgTTGAGTCGACACATCAAC | reverse primer for pCV:p126 by lic |
| PVX p25-ORF-f | cgacgacaagaccgtcaccATGGATATTCTCATCAGT | forward primer for pCV:p25 by lic |
| PVX p25-ORF-r | gaggagaagagccgtcgTGGCCCTGCGCGGACATA | reverse primer for pCV:p25 by lic |
| TuMV CP-194nt-f | AAGACCGACCATACATGCCA | forward primer for RT-PCR |
| TuMV CP-194nt-r | ACCAACGTTTCCATCCAAGC | reverse primer for RT-PCR |
| Actin-482nt-f | CCCAAAGGCTAATCGTGAAA | forward primer for RT-PCR |
| Actin-482nt-r | GCAGCTTCCATTCCAATCAT | reverse primer for RT-PCR |
| ATG5-299nt-f | cgacgacaagaccgtTGGGAAGTAAAGGGGCAG | forward primer for TRV:ATG5 by lic |
| ATG5-299nt-r | ggaggagaagagccgtcgACGTTCAGGTTCTGCACA | reverse primer for TRV:ATG5 by lic |
| ATG7-299nt-f | cgacgacaagaccgtTGGCGGATAGTGGAAGAGGA | forward primer for TRV:ATG7 by lic |
| ATG7-299nt-r | ggaggagaagagccgtcgCAACGTGTTTGTATTGAGAA | reverse primer for TRV:ATG7 by lic |
| RPN10-f | cgacgacaagaccgtcaccTTTCAATGGAGGAGGAAA | forward primer for TRV:NbRPN10 by lic |
| RPN10-r | gaggagaagagccgtcgCTTCTTCTCTTCTTTCTGTTCC | reverse primer for TRV:NbRPN10 by lic |
| RPN13-f | cgacgacaagaccgtcaccGAGTTCCCTGGTGAAGA | forward primer for TRV:NbRPN13 by lic |
| RPN13-r | gaggagaagagccgtcgTGGGATCAATTCCAAACTGA | reverse primer for TRV:NbRPN13 by lic |
| AtOE23-ORF-f | cgacgacaagaccgtcaccATGGCGTACAGTGCGTGT | reverse primer for pCV:AtOE23-mCherry by lic |
| AtOE23-ORF-r | gaggagaagagccgtcgAGCAACACTGAAAGAAGT | forward primer for pCV:AtOE23-mCherry by lic |
| 13LOX-ORF-f | cgacgacaagaccgtcaccATGGCGTACAGTGCGTGT | forward primer for pPR3-13LOX by lic |
| 13LOX-ORF-r | gaggagaagagccgtcgAGCAACACTGAAAGAAGT | reverse primer for pPR3-13LOX by lic |
| AOS-ORF-f | cgacgacaagaccgtcaccATGGCAGTACCAGCAGCA | forward primer for pPR3-AOS by lic |
| AOS-ORF-r | gaggagaagagccgtcgAGCTCTCTTCAAAGAAG | reverse primer for pPR3-AOS by lic |
| TuMV HC-Pro-ORF-f | cgacgacaagaccgtcaccATGGCAGGTGCAGCGGGAGCC | forward primer for pCV:HC-Pro-cYFP by lic |
| TuMV HC-Pro-ORF-r | gaggagaagagccgtcgTCCAACGCGGTAGTGTTTCA | reverse primer for pCV:HC-Pro-cYFP by lic |
| TuMV P3-ORF-f | cgacgacaagaccgtcaccATGGGAACAGAATGGGAGGA | forward primer for pCV:P3-cYFP by lic |
| TuMV P3-ORF-r | gaggagaagagccgtcgTTGATGAACCACCGCCTTTTCT | reverse primer for pCV:P3-cYFP by lic |
| TuMV 6K1-ORF-f | cgacgacaagaccgtcaccATGGCGAAGAGACAATCCGAG | forward primer for pCV:6K1-cYFP by lic |
| TuMV 6K1-ORF-r | gaggagaagagccgtcgCTGATGGTAGACTGTAGGTT | reverse primer for pCV:6K1-cYFP by lic |
| TuMV CI-ORF-f | cgacgacaagaccgtcaccATGACTCTCAATGATATAGAG | forward primer for pCV:CI-cYFP by lic |
| TuMV CI-ORF-r | gaggagaagagccgtcgTTGATGGTGAACTGCCTCAA | reverse primer for pCV:CI-cYFP by lic |
| TuMV 6K2-ORF-f | cgacgacaagaccgtcaccATGAACACCAGCGACATGAG | forward primer for pCV:6K2-cYFP by lic |
| TuMV 6K2-ORF-r | gaggagaagagccgtcgTTCATGGGTTACGGGTTCGG | reverse primer for pCV:6K2-cYFP by lic |
| TuMV VPg-ORF-f | cgacgacaagaccgtcaccATGGCGAAAGGTAAGAGGCAA | forward primer for pCV:VPg-cYFP by lic |
| TuMV VPg-ORF-r | gaggagaagagccgtcgCTCGTGGTCCACTGGGACGA | reverse primer for pCV:VPg-cYFP by lic |
| TuMV NIa-ORF-f | cgacgacaagaccgtcaccATGAGTAACTCCATGTTCAGAG | forward primer for pCV:NIa-cYFP by lic |
| TuMV NIa-ORF-r | gaggagaagagccgtcgTTGTGCGTAGACTGCCGTGC | reverse primer for pCV:NIa-cYFP by lic |
| TuMV NIb-ORF-f | cgacgacaagaccgtcaccATGCTGGTGATAAACACAAGCC | forward primer for pCV:NIb-cYFP by lic |
| TuMV NIb-ORF-r | gaggagaagagccgtcgCTGGTGATAAACACAAGCCT | reverse primer for pCV:NIb-cYFP by lic |
| TuMV CP-ORF-f | cgacgacaagaccgtcaccATGGCAGGTGAAACGCTTGATG | forward primer for pCV:CP-cYFP by lic |
| TuMV CP-ORF-r | gaggagaagagccgtcgCAACCCCTGAACGCCCAGTA | reverse primer for pCV:CP-cYFP by lic |
| TuMV P3N-PIPO-ORF-f | cgacgacaagaccgtcaccATGAGCATCTCCATTTTGGAAA | forward primer for pCV:P3N-PIPO-cYFP by lic |
| TuMV P3N-PIPO-ORF-r | gaggagaagagccgtcgGATAACTTTTTTTCCAAAATGGA | reverse primer for pCV:P3N-PIPO-cYFP by lic |
| Actin-f | AAGACCAGCTCATCCGTGGA | primer as internal control used for qRT-PCR |
| Actin-r | CTCATCCTATCAGCAATGCCC |  |
| NbcpSRP54-f | AGCCTGGCAAGGATTAGA | primer used for qRT-PCR |
| NbcpSRP54-r | GGTTCAGTACCCGTTTCA |  |
| NbAOCs-f | TCTTGCTGTTACCGGTGGAT | primer used for qRT-PCR |
| NbAOCs-r | AGACGGCAGATCGGGAATAC |  |
| PDF1.2-f | CTCAGAGCCACCGTTTCAAG | primer used for qRT-PCR |
| PDF1.2-r | CTAGTACAGTTTCAACGGCGG |  |
| PR3-f | ACTTTGGATGGTGCGGAAAC | primer used for qRT-PCR |
| PR3-r | CTTCCCTTGGCATGCATTGT |  |
| PR4-f | ACTACTTGGGATGCCGACAA | primer used for qRT-PCR |
| PR4-r | TTCGTCACCCTCAAGCATCT |  |
| ATG5-f | AAGCTCATACGCATTCAGGG | primer used for qRT-PCR |
| ATG5-r | GCTTCGGACCTTTGCTACCT |  |
| ATG7-f | CCAGCAGTGGAAGCAGAAGGTCTT | primer used for qRT-PCR |
| ATG7-r | GCCACCGACTTTCCCGTGTATCA |  |
| RPN10-f | TAATTTGGATCCTGAACTTG | primer used for qRT-PCR |
| RPN10-r | TCTCCTTTCTCTTGTGTAGC |  |
| RPN13-f | GTTCGTATAGGCAGGGGTGA | primer used for qRT-PCR |
| RPN13-r | GCTGGCTCCTGTATCCAAAA |  |
