# Supplementary material for: Turnip mosaic virus P1 suppresses JA biosynthesis by degrading cpSRP54 that delivers AOCs onto the thylakoid membrane to facilitate viral infection
Source: PLoS Pathog. 2021 Dec 1;17(12):e1010108. doi: 10.1371/journal.ppat.1010108 (PMC8668097; doi:10.1371/journal.ppat.1010108)
Supplement: S2 Table — (DOCX) [file ppat.1010108.s023.docx]

**S2 Table.** **Statistics to support Fig 3C.**

Percentage of AOC.1 labeling associated to TM in chloroplasts. Gold particles were counted from 4 random chloroplast profiles.

|  | Gold particles | | Percentage | Mean percentage | Relative percentage | Error | P Value *(t* test) |
| --- | --- | --- | --- | --- | --- | --- | --- |
|  | Thylakoid | Total |  |  |  |  |  |
| TRV:00 | 66 | 94 | 0.70213 | 0.74961 | 1.00000 | 0.04420 | 0.00052 |
|  | 49 | 65 | 0.75385 |  |  |  |  |
|  | 39 | 51 | 0.76471 |  |  |  |  |
|  | 42 | 54 | 0.77778 |  |  |  |  |
| TRV:cpSRP54 | 13 | 45 | 0.28889 | 0.23938 | 0.31933 | 0.06927 |  |
|  | 13 | 56 | 0.23214 |  |  |  |  |
|  | 20 | 75 | 0.26667 |  |  |  |  |
|  | 9 | 53 | 0.16981 |  |  |  |  |
