# Supplementary material for: Turnip mosaic virus P1 suppresses JA biosynthesis by degrading cpSRP54 that delivers AOCs onto the thylakoid membrane to facilitate viral infection
Source: PLoS Pathog. 2021 Dec 1;17(12):e1010108. doi: 10.1371/journal.ppat.1010108 (PMC8668097; doi:10.1371/journal.ppat.1010108)
Supplement: Attachment — Submitted filename: Response to Reviewers.docx [file ppat.1010108.s024.docx]

**Authors’ response to comments received**

We thank the editor and the reviewers for their constructive comments and have now modified the script to correct the errors and to respond to the comments and suggestions. In the text below, the comments received are in black and our replies in red.

**Part I -Summary**

Reviewer #1:

Recent studies have demonstrated that genetic and chemical manipulation of JA can significantly alter plant-virus interactions. The authors previous work demonstrated the cpSRP54 protein was significantly reduced by RSV infection in N. benthamiana. In this manuscript the authors attempt to dissect the role of JA and cpSRP54 in plant-TuMV interactions. This is an exciting and important area of research where many questions remain. The authors demonstrate here:

1. cpSRP54 protein levels are reduced in *N. benthamiana* during TuMV infection and silencing cpSRP54 increased TuMV CP levels (Fig S2; Fig 1A)

2. Silencing cpSRP54 decreased JA and JA-related transcript abundance (Fig 1A-C, Fig. S3C).

3. Spraying with MeJA decreased TuMV CP protein levels (Fig. S5)., while silencing AOC (related to JA biosynthesis) increased TuMV CP abundance (Fig 6D)

4. cpSRP5 interacted with AOC (Y2H, Co-IP, and BIFC) in *N. benthamiana* (Fig. 2) and rice and Arabidopsis (BIFC; Fig S9)

5. Full AOC localization in the thylakoid membrane requires cpSRP54 (Fig 3).

6. During TuMV infection AOC localization in protoplast is changed (Fig. 4A).

7. P1 and cpSRP54 interact (BIFC, co-IP, Y2H) (Fig. 5A,B,C)

8. P1 decreases cpSRPR54 protein levels (Fig 5D) and JA levels (Fig S12 B)

9. Other viruses (PMMoV and PVX) and their silencing suppressors reduce cpSRP54 (Fig. 6A; Fig. S16 A).

10. Silencing cpSRP54 increases CP levels for PMMoV and PVX and treating with MeJA decreases CP for these viruses.

The authors did a great job with most of the experiments and the paper advances our understanding of the importance of cpSRP54 in TuMV-plant interactions. However, I have a few concerns that need to be addressed before this manuscript is ready for publication. See comments below.

Reply: We appreciate the reviewer’s constructive comments. We have accepted the comments and revised the manuscript accordingly.

Reviewer #2:

Ji et al. discovered that TuMV P1 protein interacted with the chloroplast protein cpSRP54 and mediated its degradation through the 26S proteosome and autophagy pathways during the viral infection. This would lead to block the delivery of the allene oxide cyclases (AOCs), key Jasmonic acid biosynthesis enzymes, onto the thylakoid membrane (TM). Since JA is a crucial hormone in plant antiviral immunity, the failure of JA biosynthesis could impair plant immunity and enhance viral infection. The authors suggested that this may be a common mechanism used by viruses to counter the antiviral JA pathway. It is an interesting work, however, a few aspects should be addressed.

1. cpSRP54 responds to deliver all the thylakoid membrane proteins, including AOCs and all the photosynthesis-related proteins. Thus, its knockdown could have a great effect on the efficiency of photosynthesis. The knockdown plants showed a chlorosis phenotype as expected (shown in Fig. S2). The relative expression of cpSRP54 is about 5% left in the knockdown plants. However, using the hairpin construct to knock down the gene expression has also shown about 6% left but not chlorosis. How can this explain the relationship of this protein cpSRP54 responding to photosynthesis with the phenotype?

Reply: Thanks for the comment. The hairpin construct was expressed in the developed leaves and did not cause chlorosis. In the VIGS experiment, chlorosis appeared only on the newly emerging leaves and not on those already developed (see the figure below). We suppose that the function of cpSRP54 in chloroplast development is much more important in the developing leaves.

Fig. Phenotype of TRV:cpSRP54 treated *N.benthamiana* at 14 dpi (The inoculated leave was labeled with “a”. The developed leaves were labeled with “b”. The newly emerged leaves labeled with “c”).

2. The relative accumulation level of CP on the western blots, as an example shown in Figure 1, that using RBCL as the loading control is not a fair comparison. The phenotype of the knockdown plant is in a chlorosis phenotype that the expression level of RBCL is no longer a good marker used as a loading control.

Reply: Thank you. We have now repeated all the experiments with the commonly-used β-Actin as control [1-3]. The results are shown in the revised text as Fig 1D, Fig 1G, Fig 2J, Fig S2C, Fig S18C.

3. Because cpSRP54 responds to deliver the TM-related proteins on the membrane of TM, the effects on the reduced expression could be in all aspects. How can the authors exclude the effect derived from other proteins beyond the AOCs? Although the authors did exclude the effect derived from chlorosis and exogenous application of MeJA alleviated the susceptibility of AOC-knockdown plants to TuMV. Did the AOCs-knockdown plants shown any particular phenotype?

Reply: We thank the reviewer for pointing out this important issue. We do not think that we can completely rule out such an effect but silencing of *AOCs* did not cause any obvious phenotype. We have indeed tried to detect other possible ways in which cpSRP54 might function in defense against viruses, including the SA and ABA pathways. The SA and ABA content was not changed significantly in *cpSRP54*-silenced plants, which indicates that the SA and ABA pathways are not potentially cross talking with cpSRP54-mediated immunity (S3A and S3B Fig). We have now added a discussion to illuminate this point (Lines 349-357).

4. Line 199, “However, in cpSRP54-silenced cells, the fluorescence from AOC.1-GFP was much more diffused (Fig 3A).” The word “diffused” in the description is vague, hard to make a comparison.

Reply: We have now revised the description as “However, in *cpSRP54*-silenced cells, fewer green punctate structures of AOC.1-GFP were detected (Line 198-199).

5. The resolution of the immunolocalization under electron microscopy was not clear. It is hard to see the gold particles on the figure. Could it be replaced with a better one?

Reply: We have replaced the picture with one of higher resolution and adjusted its size to make the gold particles more visible (Fig 4B).

6. In Figure 3D, the authors indicated that the protein AOC.1-GFP in the thylakoid fraction of cpSRP54-silenced cells was much less than that in non-silenced cells. This description required control to reveal that the level of thylakoid marker was not changed in the knockdown plants. The authors used the anti-PC as the control marker. However, the signal of PC banding in the western blot is not clear. Whether using AtOE23-mCherry used in Figure 3A is better in this experiment?

Reply: Agreed. We have now repeated the experiments and analyzed the protein level of AOC.1-GFP using AtOE23-mcherry as a reference. The results are provided in the revised MS (Fig 4D).

7. The data in Figure 4C was not matched to the description in line 228 that the relative level in the infected plants declined continuously, indicating that TuMV suppressed JA biosynthesis (Fig 4C). By contrast, the level of JA in the infected plants is higher than that in the mock-inoculated plants shown in figure 4C. There must be some mistake or wrong figure used in this result.

Reply: We are sorry for the vague description causing misunderstanding. The production of JA did indeed increase significantly at the early stages of viral infection, which we suppose was the normal response of the plant to a pathogen. However, as the virus infection progressed, JA levels in the infected plants declined continuously, and reached a level significantly lower than that in the uninfected control at 9.5 dpi, which indicates that TuMV suppressed JA biosynthesis. We have now added a more detailed description in the revised text (Lines 235-239).

8. The interaction of cpSRP54 and P1 was revealed by Y2H, co-IP, and BiFC. However, the confocal result of BiFC was not clear might due to the resolution was not high enough. The results indicated that the interaction of P1-nYFP and spSRP54-cYFP was co-localized with chloroplast. Is this true shown in Figure 5A? However, the 26S proteasome is in the cytosol, how can this interaction work during TuMV infection?

Reply: Thanks for the comment. We have now provided a high-resolution figure of the BiFC, showing that the interaction between P1 and cpSRP54 occurred in chloroplasts.

We agree with the reviewer that it is difficult to understand the relationship between cytosol-localizing 26S proteasome pathway and chloroplast-localizing cpSRP54-P1. However, several reports have indicated an association between the 26S proteasome and chloroplasts. For example, some E3 ligases are localized in plastids and can directly target chloroplast protein for degradation by the 26S proteosome [5, 6]. Also, some E3 ligases can ubiquitylate chloroplast proteases, thereby regulating protein degradation in chloroplasts [7]. It therefore seems possible that cpSRP54-P1 associates with the 26S proteosome in a similar manner but the exact mechanism of interaction between cpSRP54-P1 and the 26S proteosome needs be explored next. We thank the reviewer for pointing out a key issue to better understand the interplay of cpSRP54-P1 with the 26S proteosome and have now added a paragraph to discuss it (Lines 368-378).

9. The results of treating the leaves with MG132 or 3MA that restored the level of cpSRP54 shown in Figure 5E and concluded cpSRP54 could be degraded by proteasome and autophagy pathways were vague. The virus infection or agroinfiltration could induce autophagy that targets the chloroplast (chlorophagy), the contents inside the chloroplasts (cpSRP54 might be included) were engulfed to chlorophagy for degradation in the vacuole. Therefore, blocking the autophagy pathway could also prevent the degradation of cpSRP54 in the chloroplast possibly. This hypothesis is not necessary to go through the interaction with P1.

Reply: Thanks for the comment. In this experiment, we used empty GFP as control to detect the level of cpSRP54-Myc in the presence (or absence) of MG132 or 3-MA. The results (in S14A Fig) show that MG132 or 3-MA treatment did not affect cpSRP54-Myc levels significantly, which indicates that cpSRP54 might not be targeted by the degradation system. To confirm that P1 is specifically related to cpSRP54 degradation, we have added an experiment using TuMV CP as control. This showed that CP expression did not affect the protein level of cpSRP54-Myc, whether treated with MG132 or 3-MA or not. We have now added these results in the text (Line 266, S14B Fig).

Reviewer #3: In this manuscript, Ji et al. report an interesting story of viral proteins suppress JA biosynthesis to facilitate viral infection. The authors found that viral proteins suppress JA-mediated defense by degrading cpSRP54 to inhibit the delivering of AOCs onto the thylakoid, which resulting in the decrease of JA biosynthesis. Overall, this manuscript expands on our current knowledge regarding how virus overcome the host antiviral immune response.

**Part II – Major Issues: Key Experiments Required for Acceptance**

Reviewer #1: 1. AOC interactions with cpSRP54 and localization in the thylakoid membrane needs to be shown in the viral context. AOC immunolocalization in TuMV infected plants needs to be conducted or immunoblots need to be conducted with thylakoids from mock and TuMV infected plants, probing against AOC.
Reply: We agree with the reviewer and have now added the results of BiFC assay to demonstrate the interaction between cpSRP54 and AOC in TuMV-infected plants. Moreover, AOC levels in thylakoids isolated from mock and TuMV-infected plants were also detected. These results have been added in the revised text (Lines 224-229, Fig 5B and S9 Fig).

2. AOC localization in the thylakoid membrane needs to be shown in the P1 protein context. The authors show AOC synthase accumulation is reduced in thylakoid membranes in the plants that were infiltrated with antisense cpSRP54 using immunolocalization and immunoblots, but it is very important to show the same thing happening in plants that are infected with TuMV (see comment 1) and in plants that are transiently expressing P1 only to fully support their major claims.
Reply: We agree with the reviewer on this constructive comment. We have now added the experiments to detect AOC accumulation in the context of P1 expression or viral infection. As expected, AOC delivery was blocked in these cases. The results have been added in the revised text (Fig 6E, Lines 228-229).

3. No quantitative measures of viral titer are conducted to demonstrate significant differences. Instead CP protein levels were measured, which are not quantitative, and differences in CP levels are very minor in several important figures (Fig 1, Fig S6E, Fig. S3C. Fig 6D). For example, qRT-PCR needs to be conducted for TuMV CP transcripts for the experiments in Fig 1 D to clearly demonstrate significant changes in TuMV titer or lack of. Differences in CP levels are hard to differentiate and this experiment is very important. Adding qRT-PCR data in other experiments were differences in CP protein levels were difficult to resolve would significantly strengthen the conclusions (Fig 1, Fig S6E, Fig. S3C. Fig 6D).

Reply: Agreed. We have now added qRT-PCR results to the original Fig 1, Fig S6E, Fig S3C, Fig S6D to further examine the significant changes in TuMV titer (Fig 1F, Fig 2D, Fig 2I, Fig 2K, S2D Fig, S4D Fig).

Reviewer #3: However, before acceptance for publishing in PloS Pathogens, the following concerns need to be addressed.
1. The author concluded that the decrease of JA biosynthesis due to the abnormal delivering of AOCs onto the thylakoid. They should detect the expression level of AOCs and the protein level of AOCs post infected with virus. Whether the interaction of cpSRP54 and AOCs influence the stability of AOCs.

Reply: Thanks for the comment. We have now added the experiment to detect transcript level of AOCs (AOCs protein was not detected in the absence of antibody) in TuMV-infected plants. Results showed the up-regulated expression of AOCs in TuMV-infected plants, which might be a compensation effect of loss function of AOCs protein. We have now added the results in the text (S11 Fig, Lines 231-233).

To know whether the interaction of cpSRP54 and AOCs influences the stability of AOCs, we expressed AOC.1-GFP with cpSRP54-Myc or the control GUS-Myc. Results showed that the accumulation of AOC.1-GFP was not affected by expression of cpSRP54, which indicates that the interaction of cpSRP54 and AOCs did not influence the stability of AOCs. We have now added this result in the text (S6 Fig, Lines 211-215).

2. They believed that “When AOC.1-GFP was co-expressed with cpSRP54-Myc in TuMV infected plants, the normal distribution of AOC.1 was partially restored.”. However, I cannot see any difference between cpSRP54-Myc and mock. They should provide more evidences.

Reply: Thanks for the comment. We have now provided a better image with high resolution to support the results (Fig 5C). Moreover, we added experiments isolating thylakoid to detect AOC.1 accumulation by immunoblots, which provides additional evidence. We have now added this result in Fig 5D.

3. The protein level of AOCs in the thylakoid was just detected in TRV-cpSRP54. To support the conclusion that virus inhibits the delivering of AOCs onto the thylakoid, the protein level of AOCs in the thylakoid should be also measured in virus infected and P1-Myc plants.

Reply: Agreed. We have now added these results in the text (results from virus-infected plants were added at Lines 228-229 and results from P1-myc plants were added in Fig 6E).

**Part III – Minor Issues: Editorial and Data Presentation Modifications**

Reviewer #1: 4. The claim that TuMV alters JA content is important, but details on methods are missing making Fig 4C making it difficult to interpret. How were these plants inoculated and how were “Mock” plants treated? JA is damage induced and increases in JA in early time points and decreases in late time points could be due inoculation damage if the mocks were not also damaged in the same way.
Reply: Thanks for the comment. Indeed, JA could be induced by damage. In the experiments, plants were agroinfiltrated for viral inoculation with agrobacterium containing TuMV plasmid. In the mock, the agrobacterium with empty plasmid was used for inoculation, which decreases the effect of damage on JA induction. We have now added a detailed description in the figure legends (Line 853).

5. The title should be revised to focus on P1, as the authors have only shown convincingly that P1 degrades plant protein cpSRP54 that prevents delivery of AOC to thylakoid and suppress JA biosynthesis. Although the authors show that two viral proteins p25 and p126 interacts with cpSRP54, the subsequent steps of that interaction suppressing JA biosynthesis does need to be demonstrated or the title revised.
Reply: Agreed. We have now revised the title to “Turnip mosaic virus P1 suppresses JA biosynthesis by degrading cpSRP54 that delivers AOCs onto the thylakoid membrane to facilitate viral infection”.

6. The other alternate hypothesis that is not properly addressed in the manuscript is the possibility of some other protein or mechanism that might crosstalk with cpSRP54’s removal by P1 to alter viral titer. A quick search shows cpSRP54 Arabidopsis mutants have altered photosynthesis, ROS production, ABA, and carotenoids. Is it possible one of these changes may be mediating impacts on plant- virus interaction and may be more important for cpSRP54-TuMV interactions?
Reply: Thanks for the comment. In fact, in the experiments, we also detected ABA and SA content in *cpSRP54*-silenced plants. However, we found that ABA and SA content was not affected significantly in silenced *N. benthamiana* and so we did not focus further on them. However, we accept that we cannot completely rule out the possibility that there may be other effects of cpSRP54 that crosstalk with the cpSRP54-AOC interaction. We have now added some discussion on this in the text (Lines 349-357).

7. Is it possible that removal of cpSRP54 may hamper JA biosynthesis at a step before AOC synthase or after it? I think that measuring JA, AOC thylakoid localization, or possibly the substrate for AOC synthase in plants that were infiltrated with inhibitors of proteasome and autophagy or in the RPN11/ATG5, AT7 silenced plants, expressing cpSRP54 and PI may be a definitive step towards showing that P1 degrades cpSRP54, which stops AOC synthase movement and that is the exact step which is targeted by the viral effector to reduce JA biosynthesis.

Reply: We agreed the reviewer on the constructive comment. We have now added the experiments to detect JA content and AOC thylakoid localization in the presence of P1 in *RPN10*-silenced plants. Silencing of *RPN10* restored the normal thylakoid distribution of AOC.1 and alleviated the reduced JA levels caused by P1 expression, which supports our conclusion. We have now added these results in the revised text (lines 271-275).

8. Some of the figures in supplemental are quite important and should be moved to the main text of the manuscript. For example, Fig S2, Fig. S5, Fig S6a-D and S12 B should be in the main text. The results section in lines 149 to 180 solely relies on supplemental figures which shows how important some of these figures are that are in supplemental and should be moved to the main text.
Reply: Agreed. The original Fig S2, Fig. S5, Fig S6a-D and S12 B have now been moved in the main text.

9. The methods section is a bit too terse. Please mention important details such as how many biological replicates were used for each expt. This information is sporadically given for the experiments performed. How many plants were used for real time PCRs? What were their age? How many biological replicates were used for the immunoblot analyses. How many times were the BiFC experiments repeated/done? The images shown are from one plant, but did the authors confirm the same result in different biological replicates? If so, how many? Please give more details about what plasmids were used for the BiFC constructs.

Reply: Thanks for the comment. We have now added this information in the Materials and Methods section and figure legends (Lines 413-414, 416-430, 773-774, 786-787, 791, 799-780, 804-805, 847-848, 852, 875, 884-885, 911-912, 922-923, 931, 964-965, 978-979).

1.	Yang Y, Fan G, Zhao Y, Wen Q, Wu P, Meng Y, et al. Cytidine-to-Uridine RNA Editing Factor NbMORF8 Negatively Regulates Plant Immunity to Phytophthora Pathogens. Plant Physiol. 2020;184(4):2182-98. Epub 2020/09/26. doi: 10.1104/pp.20.00458. PubMed PMID: 32972981; PubMed Central PMCID: PMCPMC7723075.

2.	Hristou A, Gerlach I, Stolle DS, Neumann J, Bischoff A, Dünschede B, et al. Ribosome-Associated Chloroplast SRP54 Enables Efficient Cotranslational Membrane Insertion of Key Photosynthetic Proteins. Plant Cell. 2019;31(11):2734-50. Epub 2019/08/25. doi: 10.1105/tpc.19.00169. PubMed PMID: 31444312; PubMed Central PMCID: PMCPMC6881123.

3.	Liu R, Xu YH, Jiang SC, Lu K, Lu YF, Feng XJ, et al. Light-harvesting chlorophyll a/b-binding proteins, positively involved in abscisic acid signalling, require a transcription repressor, WRKY40, to balance their function. J Exp Bot. 2013;64(18):5443-56. Epub 2013/10/01. doi: 10.1093/jxb/ert307. PubMed PMID: 24078667; PubMed Central PMCID: PMCPMC3871805.

4.	Yu B, Gruber MY, Khachatourians GG, Zhou R, Epp DJ, Hegedus DD, et al. Arabidopsis cpSRP54 regulates carotenoid accumulation in Arabidopsis and Brassica napus. J Exp Bot. 2012;63(14):5189-202. Epub 2012/07/14. doi: 10.1093/jxb/ers179. PubMed PMID: 22791829; PubMed Central PMCID: PMCPMC3430994.

5.	Ling Q, Broad W, Trösch R, Töpel M, Demiral Sert T, Lymperopoulos P, et al. Ubiquitin-dependent chloroplast-associated protein degradation in plants. Science. 2019;363(6429). Epub 2019/02/23. doi: 10.1126/science.aav4467. PubMed PMID: 30792274.

6.	Ling Q, Sadali NM, Soufi Z, Zhou Y, Huang B, Zeng Y, et al. The chloroplast-associated protein degradation pathway controls chromoplast development and fruit ripening in tomato. Nat Plants. 2021;7(5):655-66. Epub 2021/05/20. doi: 10.1038/s41477-021-00916-y. PubMed PMID: 34007040.

7.	Shen G, Adam Z, Zhang H. The E3 ligase AtCHIP ubiquitylates FtsH1, a component of the chloroplast FtsH protease, and affects protein degradation in chloroplasts. The Plant Journal. 2007;52(2):309-21. doi: <https://doi.org/10.1111/j.1365-313X.2007.03239.x>.
