# Supplementary material for: Turnip mosaic virus P1 suppresses JA biosynthesis by degrading cpSRP54 that delivers AOCs onto the thylakoid membrane to facilitate viral infection
Source: PLoS Pathog. 2021 Dec 1;17(12):e1010108. doi: 10.1371/journal.ppat.1010108 (PMC8668097; doi:10.1371/journal.ppat.1010108)
Supplement: Attachment — Submitted filename: response.docx [file ppat.1010108.s025.docx]

**Authors’ response to comments received**

We thank the editors and the reviewers for their constructive comments and have now modified the script to correct the errors and to respond to the comments and suggestions. In the text below, the comments received are in black and our replies in red.

Comments from the Editors:

Thank you very much for submitting your manuscript "Turnip mosaic virus P1 suppresses JA biosynthesis by degrading cpSRP54 that delivers AOCs onto the thylakoid membrane to facilitate viral infection" for consideration at PLOS Pathogens. As with all papers reviewed by the journal, your manuscript was reviewed by members of the editorial board and by several independent reviewers. The reviewers appreciated the attention to an important topic. Based on the reviews, we are likely to accept this manuscript for publication, providing that you address the reviewer's comments.

Specifically, the revised manuscript should include a time course data on cpSRP54 transcript level and JA content after TuMV infection. Other comments could be addressed by editing the text.

Reply: We thank the editors for their useful comments. We have now added the time course results requested. We have also added other experiments requested by the reviewers and edited the text accordingly.

Reviewer Comments (if any, and for reference):

Reviewer's Responses to Questions

**Part I - Summary**
Please use this section to discuss strengths/weaknesses of study, novelty/significance, general execution and scholarship.

Reviewer #3: This is an improved version and has addressed all my concerns. I do not have further comments.

Reviewer #4: The manuscript entailed “Turnip mosaic virus P1 suppresses JA” by Ji et al reported their discovery that TuMV infection inhibits the accmulation of cpSRP54，a chloroplast signal recognition particle subunit which is required for photosynthetic proteins to target in to thylakoid membranes. Further they showed that cpSRP54 interacts with allene oxide cyclases(AOCs) which participates in JA biosynthesis. Interestingly, they demonstrated that TuMV P1 interacts with cpSRP54 and mediated the degradation of cpSRP54 via 26S proteasome and results in the reduction of JA synthesis, further benefits viral infection. More interestingly, they showed evidences that this strategy may be applied by different RNA viruses such as PVX and TMV. The story is very interesting and the figures are well illustrated. The two reviewers have raised a number of reasonable questions, and the authors have substantially addressed their concerns. However, after carefully read the manuscript, I still have some questions and there are some minor errors should be corrected before acceptance for publication. I have listed below.

Reviewer #5: In this article, Ji et al. have described an interesting viral counter-defence strategy to overcome antiviral JA response. This study is interesting and will add on to our current understanding of plant defence and viral counter-defence. However, there are some major and minor concerns which need to be addressed before the manuscript is accepted for publication.

**Part II – Major Issues: Key Experiments Required for Acceptance**
Please use this section to detail the key new experiments or modifications of existing experiments that should be absolutely required to validate study conclusions.

Generally, there should be no more than 3 such required experiments or major modifications for a "Major Revision" recommendation. If more than 3 experiments are necessary to validate the study conclusions, then you are encouraged to recommend "Reject".

Reviewer #3: (No Response)

Reviewer #4: Major: I would happy to see if the authors could add a time course of results of JA content corelated with cpSRP54 and AOCs expressions after TuMV infection.

Reply: We have now added the results in the revised manuscript. The JA content increased at early stages of viral infection, but then declined steadily, reaching its lowest level at 9.5 dpi, indicating the ability of TuMV to suppress JA biosynthesis (Fig 5D). At the later stages of TuMV infection, JA levels increased as the plants reacted to maintain a JA balance. Consistent with this, the expression levels of *cpSRP54* and *AOCs* showed a similar pattern (S1C and S11 Fig). We have added these results in the text (Lines 120-121, 235-236) and discussed it briefly (Lines 395-397, 406-409).

Reviewer #5: (1) Authors need to check JA level beyond 9.5 days post inoculation (dpi) also (at least up to 21 dpi).

Reply: Thanks for the comment. We have now added the results showing JA levels in samples at 11, 12.5 and 14 dpi. Virus-infected plants had obvious necrosis or were dead at 15.5 dpi so we did not collect samples beyond that time point. The results have been added and discussed (Fig 5D. Lines 242-244, Lines 406-409).

(2) Author need to provide information on check the cpSRP54 transcript abundance during TuMV pathogenesis at different dpi.

Reply: Agreed and done as explained above. The time course results have been added (Lines 120-121) and discussed (Lines 395-397).

(3) Expression of other JA biosynthetic genes which function upstream of AOCs (specifically AOS and LOX genes) should be checked in cpSRP54 silenced plants.

Reply: We have now added qRT-PCR experiments that show that transcripts of *13LOX* and *AOS* genes in *cpSRP54*-silenced plants were down-regulated (Lines 364-367, S20 Fig).

(4) What is the subcellular localization of TuMV encoded P1 protein?

Reply: We have now performed a confocal microscopy analysis of transiently expressed P1-GFP in *N.benthamiana* leaves. This showed that P1-GFP localized in both the cytoplasm and the nucleus, which is consistent to a previous molecular characterization study of P1 (Pan. Molecular Characterization of the Potyviral First

Protein (P1 Protein). 2016). We have added these results in the revised text (S21 Fig, Lines 379-380).

It has been reported that P1 contains two domains. In our subcellular localization analysis, both the conserved C-terminal protease domain (P1C) and the hypervariable N-terminal portion (P1N) were present in both the cytoplasm and the nucleus (like full-length P1), but P1N was also localized in chloroplasts (see the figure below), which indicates a potential function of P1 in chloroplasts. Our BiFC results showed that the P1-cpSRP54 complex was also present in chloroplasts (Fig 6A), which we suppose may induce or amplify the chloroplast-related effect of P1.

Subcellular localization analysis of transiently expressed GFP, P1-GFP, P1N-GFP and P1C-GFP in *N.benthamiana*. Bars, 10 µm.

(5) Authors must put effort to identify the specific domain or amino acid stretch of P1 protein responsible for the interaction with cpSRP54. This reader is of the opinion that authors must express a p1 mutant unable to interact with cpSRP54 and validate their observation by comparing with the wild-type viral protein.

Reply: We agree with the reviewer about this suggestion. Over the last month, we have done experiments to identify the possible domain of P1 interacting with cpSRP54. Based on the predicted domains of P1, we expressed 5 truncated P1 proteins and detected their interaction with cpSRP54 by BiFC. Unfortunately, all the proteins still interacted with cpSRP54 (see figure below) and we therefore have no idea how to perform the suggested control experiments at present. We will keep this suggestion in mind and validate the observation when the structure of P1 protein is better understood.

Interaction of 5 truncated P1 proteins with cpSRP54 by BiFC. Bars, 10 µm.

(6) It will be interesting to know the impact of the interaction between cpSRP54 and AOC.2 in TuMV pathogenesis. Whether it follows the trend of AOC.1 or not needs to be investigated.

Reply: Thanks for the comment. We have now added the results in the text (Lines 264-266). As expected, AOC.2 delivery was blocked in the context of P1 expression or TuMV infection.

**Part III – Minor Issues: Editorial and Data Presentation Modifications**
Please use this section for editorial suggestions as well as relatively minor modifications of existing data that would enhance clarity.

Reviewer #3: (No Response)

Reviewer #4: Minor:

1. Some information for Ref. 21 and 22 is missing.

Reply: Corrected.

2. There is a most recent review paper about the interactions between host hormones and plant viral infection should be cited. See PLoS Pathogens 2021,17(2):e1009242.

Reply: Thanks. We have now added this citation.

3. Some figure legends are not matched with figures, such as Fig. 1C, 1D, 1F and 1G. Please check them carefully.

Reply: Corrected.

4. The model, some steps such as JA activates which defense genes? How cpSRP54 exits from chloroplast and be degraded is not fully understood. Therefore, the lines should use dashed lines.

Reply: Thanks for the suggestion. We have specified the defense genes and changed the lines to dashed lines.

5. Some paragraph on the introduction of cpSRP54 should be more to introduction.

Reply: We have now added a sentence to introduce cpSRP54 in the Introduction (Lines 96-98) ahead of more detailed comments in the Discussion.

Reviewer #5: (1) Line 25: biosynthesis should also be mentioned here along with signaling.

Reply: Added.

(2) Line 28-29: Intended meaning not clear!

Reply: We deleted “which indicated impaired immunity”.

(3) Line 73, 82: rice infecting viruses NOT of rice viruses.

Reply: Changed as suggested.

(4) Line 90: ‘and’ would be more suitable word than ‘or’ here.

Reply: Changed.
